# Evolutionary Constraints in Hind Wing Shape in Chinese Dung Beetles (Coleoptera: Scarabaeinae)

**DOI:** 10.1371/journal.pone.0021600

**Published:** 2011-06-27

**Authors:** Ming Bai, Erin McCullough, Ke-Qing Song, Wan-Gang Liu, Xing-Ke Yang

**Affiliations:** 1 Key Laboratory of Zoological Systematics and Evolution, Institute of Zoology, Chinese Academy of Sciences, Beijing, People's Republic of China; 2 Division of Biological Sciences, University of Montana, Missoula, Montana, United States of America; University of Chicago, United States of America

## Abstract

This study examines the evolution hindwing shape in Chinese dung beetle species using morphometric and phylogenetic analyses. Previous studies have analyzed the evolution of wing shape within a single or very few species, or by comparing only a few wing traits. No study has analyzed wing shape evolution of a large number of species, or quantitatively compared morphological variation of wings with proposed phylogenetic relationships. This study examines the morphological variation of hindwings based on 19 landmarks, 119 morphological characters, and 81 beetle species. Only one most parsimonious tree (MPT) was found based on 119 wing and body characters. To better understand the possible role of the hindwing in the evolution of Scarabaeinae, additional phylogenetic analyses were proposed based on the only body features (106 characters, wing characters excluded). Two MPT were found based on 106 body characters, and five nodes were collapsed in a strict consensus. There was a strong correlation between the morphometric tree and all phylogenetic trees (r>0.5). Reconstructions of the ancestral wing forms suggest that Scarabaeinae hindwing morphology has not changed substantially over time, but the morphological changes that do occur are focused at the base of the wing. These results suggest that flight has been important since the origin of Scarabaeinae, and that variation in hindwing morphology has been limited by functional constraints. Comparison of metric disparity values and relative evolutionary sequences among Scarabaeinae tribes suggest that the primitive dung beetles had relatively diverse hindwing morphologies, while advanced dung beetles have relatively similar wing morphologies. The strong correlation between the morphometric tree and phylogenetic trees suggest that hindwing features reflect the evolution of whole body morphology and that wing characters are suitable for the phylogenetic analyses. By integrating morphometric and cladistic approaches, this paper sheds new light on the evolution of dung beetle hind wings.

## Introduction

The evolution of flight has led to a wide variety of morphological adaptations in such flying animals as birds, bats, and insects. Studying the evolution of flight characters is important to understanding the different selective external forces that have shaped the size and shape of wings and other flight traits, and how these adaptations may be limited by developmental or phylogenetic constraints [1], [2]. For example, several studies have demonstrated that wing shape in birds is affected by migration distance [3], [4], [5], sexual selection [6], [7], and foraging strategies [3], [8], and that flight characters, such as tail shape, can be limited by mechanical and physiological constraints [9]. In insects, as well, wing shape is likely to be affected by different selective external forces [10], [11], but the dominant drivers of wing shape evolution are generally unknown. Wing venation, folding patterns, and other wing characters have long been recognized as important in taxonomic and phylogenetic analyses [12], [13], [14], [15], [16], [17]. However, if wing characters are suitable for the phylogenetic analyses and how wing characters have evolved over time are largely unexplored.

**Figure 1:**
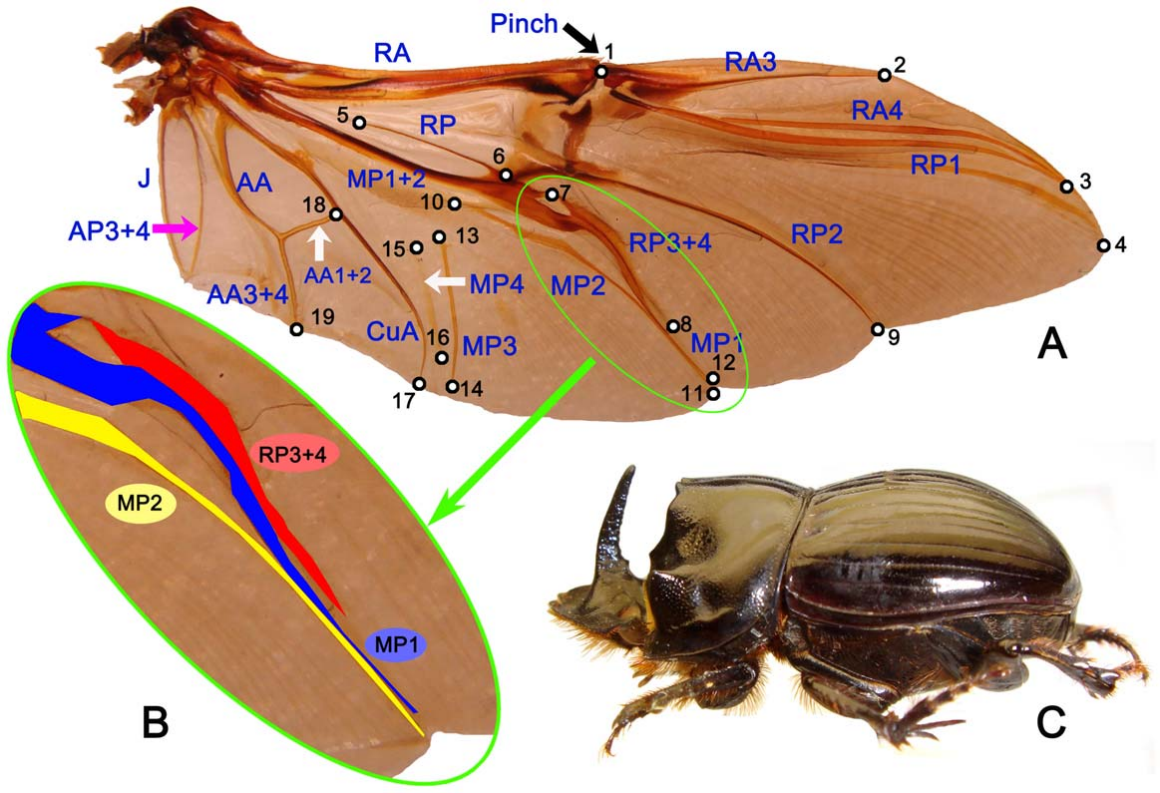
Description of the landmarks (right hind wing of *Copris lunaris* (Linnaeus, 1758)). (A) Landmark positions used in morphometric analyses. (B) Positions of RP_3+4_, MP_1_ and MP_2_, green circle in Fig. 1A. (C) Lateral view of *Copris lunaris* (Linnaeus, 1758).

Previous studies have considered the evolution of wing shape by analyzing a single or very few species [18], [19], [20], [21], [22], or by comparing only a few wing traits [23], [24] using a traditional comparative morphology approach. No study has analyzed wing shape evolution of a large number of species, or quantitatively compared morphological variation of wings in a phylogenetic context. This study examines the morphological variation of beetle hind wings based on 81 beetle species (Table s1), 19 wing landmarks (Figure 1A–B), and 119 wing and body morphological characters (Table S2, S3, Figures S1, S2, S3, S4, S5, S6, S7, S8), which were selected from Philips *et al*. [25] and coded for the Chinese dung beetles for the first time. To clarify the role of the hindwing in the evolution of the Scarabaeinae, additional phylogenetic analyses were proposed when both wing and body characters were included (119 chracters) and when only body features were used (106 characters). Specifically, this study evaluated the phylogenetic relationships of 81 dung beetle species, and analyzed the variation in hind wing morphology using morphometric approaches. The aim of this study was to compare how the evolution wing morphology is affected by whole-body morphology.

Dung beetles (Coleoptera: Scarabaeinae) are ideal organisms for studying the evolution of wing shape (Figure 1C). The dung beetles comprise nearly 6,000 described species grouped into 240 genera [26], and exist on every continent except Antarctica. As a result, they have adapted to a variety of different habitats, and exhibit a wide diversity in both wing and body morphology [23], [27], [28], [29]. Furthermore, dung beetles are one of the best-studied groups of insects in terms of ecology [30], natural history [31], behavior [32], and taxonomy and phylogeny [23], [25], [33], [34], [35], [36], so the evolutionary relationships between many dung beetle species are relatively well-established.

Remarkably, no previous phylogenetic studies have included Chinese taxa. With a landmass of 9,600,000 sq km, China is the third largest country in the world, and has a rich diversity of habitats and climates. Additionally, China comprises the transition zone of the Palaearctic and Oriental regions, and therefore exhibits an impressive diversity of beetle fauna. The principal goal of this study is not to infer the phylogeny of Scarabaeinae worldwide, but to contribute to the understanding of evolutionary relationships among Scarabaeinae using Chinese species. By integrating morphometric and clastistic analyses and comparing morphological variation in wings with hypothesized phyologenies, this paper sheds new light on the evolution of hind wings in scarab beetles.

## Results

### Phylogenetic relationships among Chinese dung beetles based on wing and body features or only body features morphological characters

**Figure 2:**
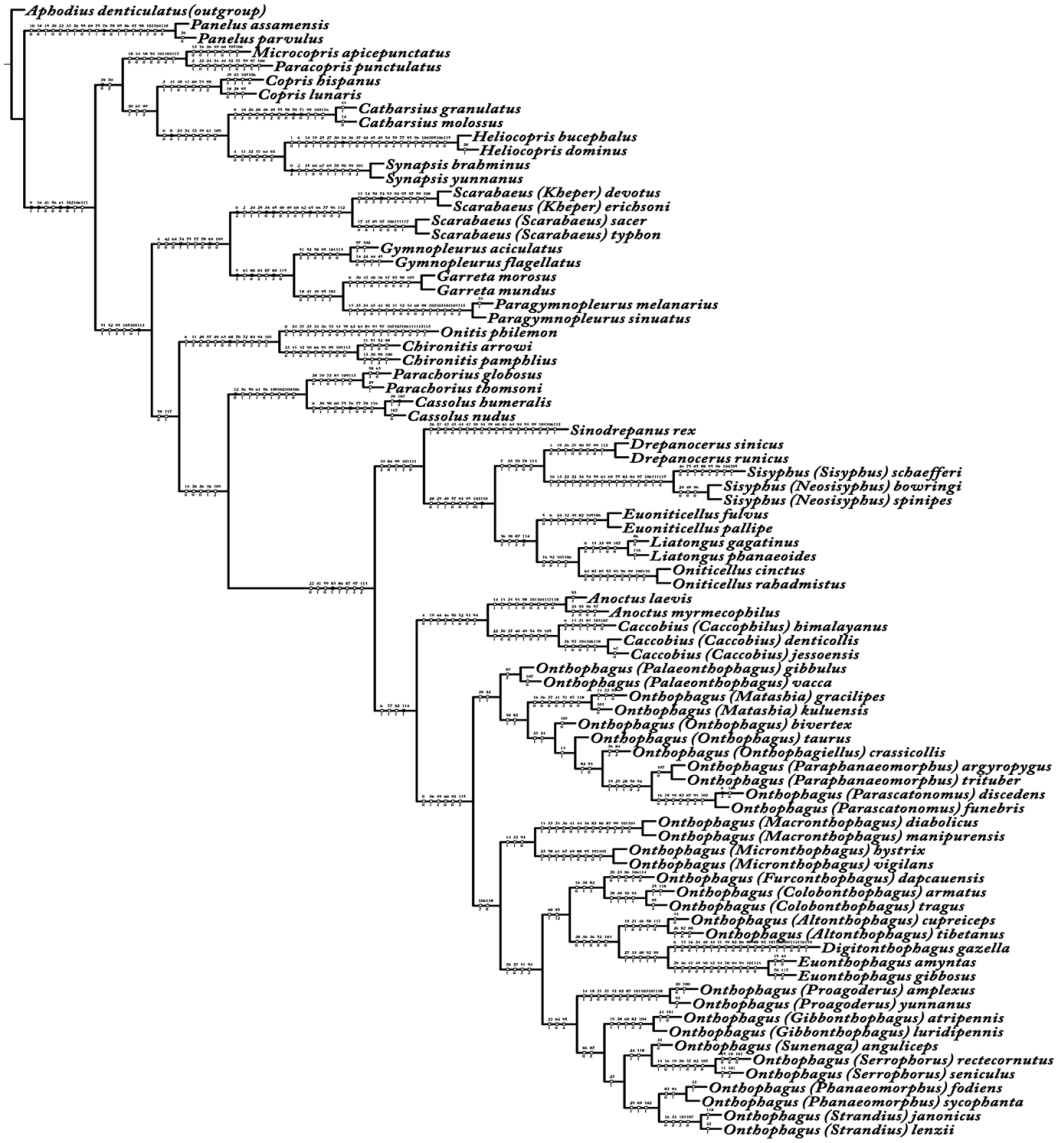
The only most parsimonious tree computed from NONA based on 119 characters. Black circles indicate nonhomoplasious changes, white circles indicate changes in homoplasious characters. Number above branches indicate character numbers, below branches indicate character states. Tree length = 965 steps, CI = 0.20, RI = 0.72.

**Figure 3:**
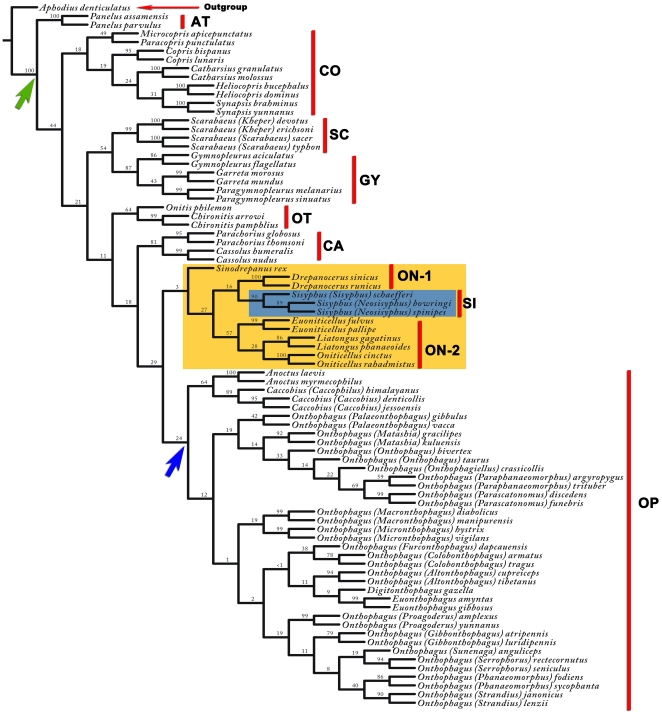
Bootstrap support for the only most parsimonious tree computed from NONA based on 119 characters. Numbers below branches indicate 1000 bootstrap support values. Tree length = 965 steps, CI = 0.20, RI = 0.72. Green arrow indicates the monophyly of Scarabaeinae. Blue arrow indicates the monophyly of Onthophagini. Brown and blue boxes indicate the relationships of Oniticellini (ON) and Sisyphini (SI).

Phylogenetic tree searches were conducted through parsimony analysis using NONA [37] software packages based on the wing and body features (119 characters) or only body features (106 characters, wing characters excluded). Broadly similar topologies were obtained from both analyses (Figure 2–3, S9, S10). Only one most parsimonious tree (tree length = 965 steps, CI = 0.20, RI = 0.72) was found based on the 119 characters. The monophyly of Scarabaeinae (green arrow in Figure 3, bootstrap = 1000) and nine tribes were well supported. The monophyly of Onthophagini (blue arrow in Figure 3), the biggest and most advanced tribe in Scarabaeinae, was supported by a total of four apomorphies: (1) [6∶1]; (2) [77∶1]; (3) [82∶1]; (4) [114∶1]. Two most parsimonious trees (tree length = 854 steps, CI = 0.20, RI = 0.72) were found based on the 106 body characters (wing characters excluded), and five nodes were collapsed in a strict consensus (Figure S9, S10).

### Morphological variations of the hind wing based on morphometric analyses

Morphological variation in hind wings was analyzed using tps-SMALL [38] based on 19 wing landmarks (Figure 1), which reflect variation in the entire hind wing of Scarabaeinae. Morphometric analyses found a strong correlation between the tangent shape and shape space. The correlation between the tangent space (Y) regressed onto Procrustes distance was 0.999988.

**Figure 4:**
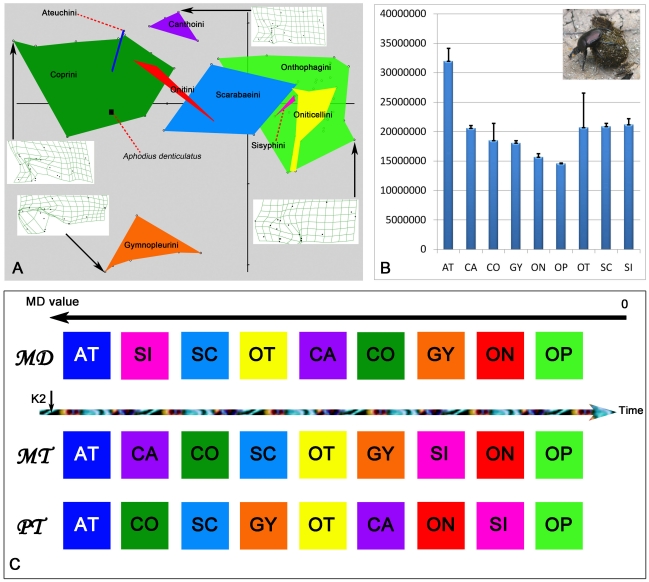
Morphological variation of hind wings based on landmark data. (A) Relative warps computed from the landmark data set. Splines indicate deformation of the landmarks in comparison to the reference configuration (Scarabaeinae, situated at the origin). (B) Metric disparity of landmark data for each tribe. Standard errors are generated from 10,000 bootstrap pseudoreplicates. (C) Comparison of metric disparity values and relative evolutionary sequence among Scarabaeinae tribes. (MD = metric disparity, MT = morphometric tree, PT = phylogenetic tree, K2 = late Cretaceous, proposed origin of Scarabaeinae).

**Figure 5:**
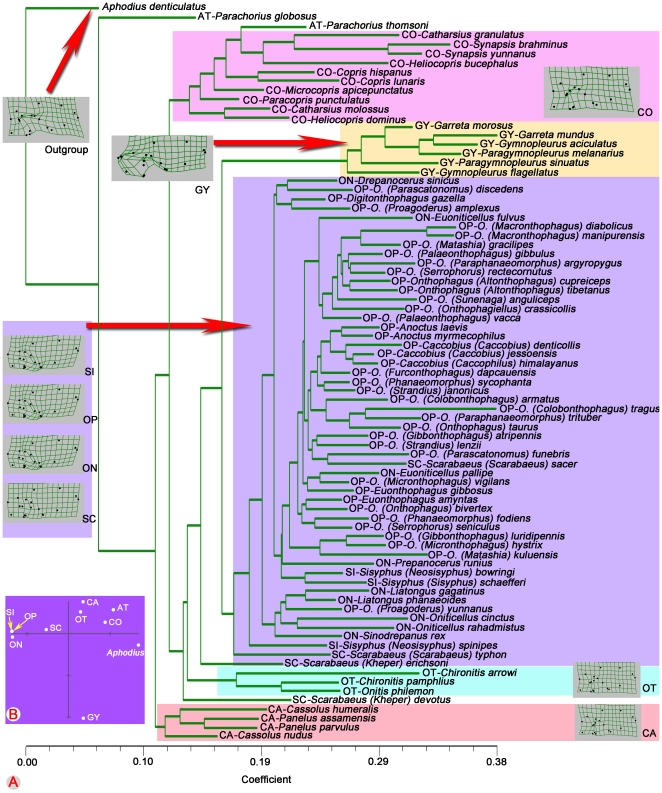
Phenetic tree of Scarabaeinae hind wing landmarks. (A) Phenetic tree based on Procrustes distances among the 81 species. (B) Shape differences of the nine Scarabaeinae tribes.

The first two relative warps of the wing landmarks accounted for 59.26% of the variation among species. These were computed by a singular value decomposition of the weight matrix [39]. The first two relative warps were plotted to indicate variation along the two axes (Figure 4A). The change in hind wing shape among species is indicated by variation along the first two relative warp axes, and shown as deformations of the least squares reference using thin-plate splines (Figure 4A). The splines show the deformation of the landmarks compared to the reference wing, computed by tps-RELW 1.44 [40], with the most significant deformation in wing landmarks having splines situated furthest from the origin. The morphological variation of each tribe is assessed quantitatively by the metric disparity (Figure 4B; Table s4), which compares the first two relative warps of the landmarks. A phenetic tree of the 81 studied dung beetle species was created from the Procrustes distance matrix (Figure 5A). The shape means for the nine tribes were plotted along the two canonical varieties axes based on the Procrustes distance matrix (Figure 5B). The splines of the tribe means and outgroup were mapped onto the phenetic tree.

### Combined analyses

#### Reconstruction of ancestral forms of hind wings of Scarabaeinae

**Figure 6:**
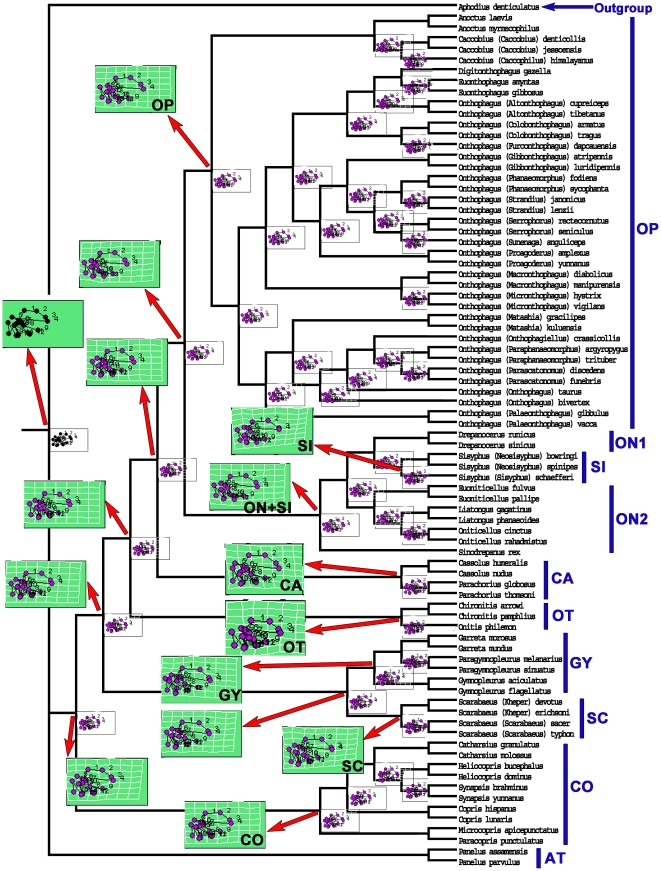
Reconstruction of ancestral forms of hind wings of Scarabaeinae.

The ancestral forms of Scarabaeinae hind wings were reconstructed by combining the wing landmark data with the only tree from the phylogentic analysis in NONA based on the 119 characters. The ancestral forms of all nodes were reconstructed using the landmark drawings module of the Rhetenor package in Mesquite [41]. The ancestral hind wing morphologies of all tribes and selected nodes are shown as magnified splines (Figure 6).

In general, the Scarabaeinae hind wing morphology has not changed substantially over the evolution of dung beetles. In particular, the R and M veins, which are likely to be important during flight by stabilizing the radial and apical wing fields [42], have been relatively stable during dung beetle evolution. These results suggest that flight has been important to dung beetles throughout their evolutionary history, and that the evolution of hind wing morphology may be limited due to functional constraints. However, most of the morphological variation that does occur among tribes was focused at the base of the hind wing, which may reflect that basal wing regions are less important during flight.

The metric disparity values from the morphometric analyses and the relative evolutionary sequence among Scarabaeinae tribes from the phylogenetic analyses (Figure 4C) suggest that the hind wing shape of primitive dung beetles was quite diverse. Although the exact evolutionary sequence of tribes cannot be reconstructed, and some tribes may have arisen at the same time, the relative evolutionary sequence of the nine Scarabaeinae tribes can be inferred from the morphometric tree (Figure 5A) and phylogenetic tree (Figure 3).

In contrast to the diverse hind wing shapes of the ancestors of the nine Scarabaeinae tribes, the wing shape of advanced dung beetles is quite similar. Given the monophyletic origin of the Scarabaeinae, these results suggest that hind wing shape has converged in surviving lineages due to similar selective forces. Indeed, most dung beetles are distributed in the lowlands of China, which supports the hypothesis that extant dung beetles have similar wing morphologies because they experience similar environmental conditions. However, more paleoecological information will be necessary to determine the environmental conditions of the ancestral dung beetles, and whether these species were subjected to more diverse selective external forces.

#### Phylogenetic and morphometric tree comparison

**Figure 7:**
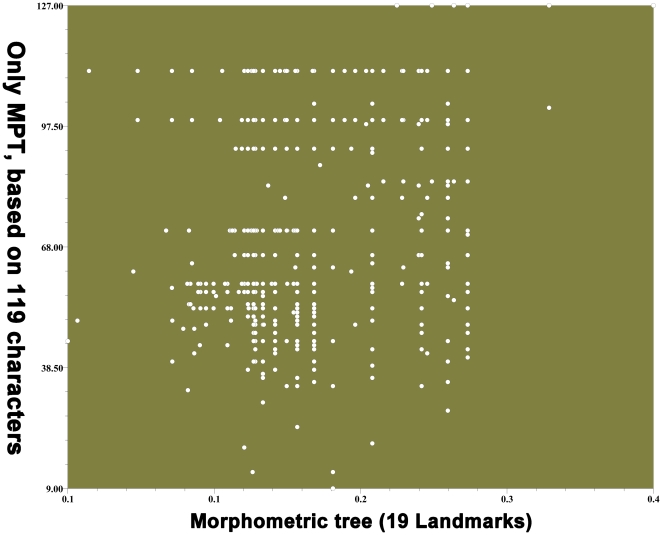
Correlation analysis of morphometric tree and the only most parsimonious phylogenetic tree based on 119 characters. Matrix correlation: r = 0.63791, significantly correlated at the 1% level.

The topologies of the trees created from phylogenetic and morphometric analyses were quite similar. There was a strong correlation (r = 0.63791) between the morphometric tree and the only most parsimonious tree based on the 119 wing and body characters (Figure 7, Table s5). There was also a good fit (r = 0.53999, 0.54107) between the morphometric tree and the other two most parsimonious trees based on the 106 body characters (Figure s11, Table s5). (Matrix correlations (r) greater than 0.5 are statistically significant at the 1% level [43]). The strong correlation between the morphometric tree and phylogenetic tree suggests that variation in hind wing shape is adequately represented by the wing features used in this study, that hind wing features reflect the evolution of whole body morphology, and that wing characters are suitable for phylogenetic analyses. As flight has likely played a key role in the radiation of insects, wings may have contributed significantly to the diversification of entire body features.

### Correlations between hind wing and body morphology

**Figure 8:**
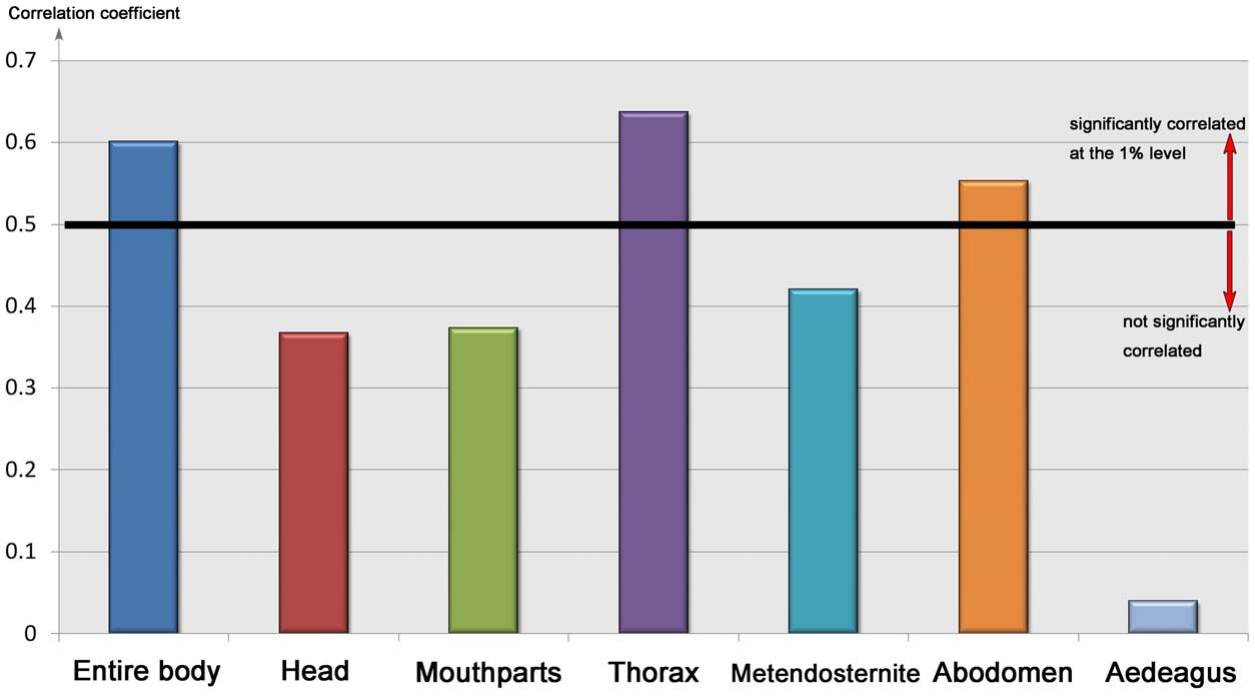
Correlation analyses between hind wing and body features.

Organisms exist as multi-trait entities, and the evolution of body features are likely to co-vary with other body parts. This study investigated the correlations between wing morphology and numerous body features, including the head, mouthparts, thorax, metendosternite, ventrites and aedeagus (Figure 8, Table S2, S3). The hind wings of Scarabaeinae were significantly correlated with the morphology of the thorax (r = 0.63663) (Table s2, character No. 61-79), abdomen (r = 0.55276) (Table s2, character No. 109-116), and entire body (r = 0.60056) (Table s2, character No. 0-118). (Matrix correlations (r) greater than 0.5 are statistically significant at the 1% level [43]). The head (r = 0.36702) (Table s2, character No. 0-7), mouthparts (r = 0. 3732) (Table s2, character No. 8-60), metendosternite (r = 0. 41987) (Table s2, character No. 93-108), and aedeagus (r = 0. 03914) (Table s2, character No. 117-118) were weakly correlated with the hind wings. These weak correlations may reflect different selective external forces between wings and the head, mouthparts and aedeagus. This hypothesis seems unlikely given that there were strong correlations between hind wing morphology and entire body features. An alternative explanation is that the morphology of the mouthparts and aedeagus are functionally constrained by the mechanics of copulation and effective food manipulation, while the morphology of wings is more evolutionarily labile.

## Discussion

This study is the first to compare variation in wing morphology among a large number of species and using a large number of morphological characters. By integrating morphometric and cladistic approaches, this paper has sheds new light on the evolution of dung beetle hind wings.

The earliest classifications and first attempts at reconstructing the evolutionary history of the Scarabaeinae was largely narrative and highly speculative [44]. Early studies [45], [46], [47], [48] were hampered by considered only a few taxa, or by examining only a limited suite of morphological characters. More recently, evolutionary relationships among the Scarabaeinae have been proposed using morphological data [25] or molecular data [49], [50], [51], although the number of taxa considered in these studies are generally still quite limited. For example, Philips *et al*. [25] hypothesized the phylogenetic relationships among 50 species of the Scarabaeinae based on 200 morphological characters, although this represents only a small subset of the nearly 6,000 described Scarabaeinae species. Furthermore, Philips *et al*. [25] did not consider the hindwing. Previous studies have considered the evolution of wing shape by analyzing a single or very few species, or by comparing only a few wing traits using a traditional comparative morphology approach, but no study has analyzed wing shape evolution of a large number of species, or quantitatively compared morphological variation of wings in a phylogenetic context. Additionally, no phylogenetic studies have included Chinese taxa, even though China encompasses an impressive landmass (9,600,000 sq km), and is the home to a large number of highly diverse dung beetle species. This paper analyzes the evolution of hind wings in the Scarabaeinae based on 19 hind wing landmarks, 119 morphological characters, and 81 species. It is the first to employ large-scale sampling and rigid quantitative analyses in order to reconstruct the evolution of wings among Chinese Scarabaeinae species.

The results of the morphometric and phylogenetic analyses suggest that wing features hind wing features reflect the evolution of whole body morphology. Furthermore, the morphological stability of the radial and apical fields (i.e. R and M veins) suggest that flight has been important since the origin of Scarabaeinae, and that variation in hind wing morphology may have been limited by functional constraints. Interestingly, reconstructions of the ancestral wing forms suggest that the hind wing morphologies of primitive dung beetles were substantially more diverse than the wing morphologies of advanced species. Future research should explore the selective external forces leading to the convergence of hind wing morphology in extant beetles, and potential developmental mechanisms driving hind wing evolution. Although previous studies have examined how ecological and environmental factors may lead to wing reduction or wing loss [52], this study is the first to explore how selective external forces may influence the lead to quantitative changes in a fully developed wing. This study makes an important contribution to our understanding of the evolutionary relationships among this species-rich insect lineage.

## Methods

This study was based on 81 species housed in the Institute of Zoology, Chinese Academy of Sciences. The specimens were dissected and examined using a LEICA MZ 12.5 dissecting microscope. Terminology used throughout this paper follow Kukalová-Peck & Lawrence [24]. Abbreviations for the tribe names used in the figures are as follows: OP = Onthophagini, CA = Canthoini, ON = Oniticellini, CO = Coprini, OT = Onitini, GY = Gymnopleurini, SC = Scarabaeini, SI = Sisyphini, AT = Ateuchini.

**Figure 9:**
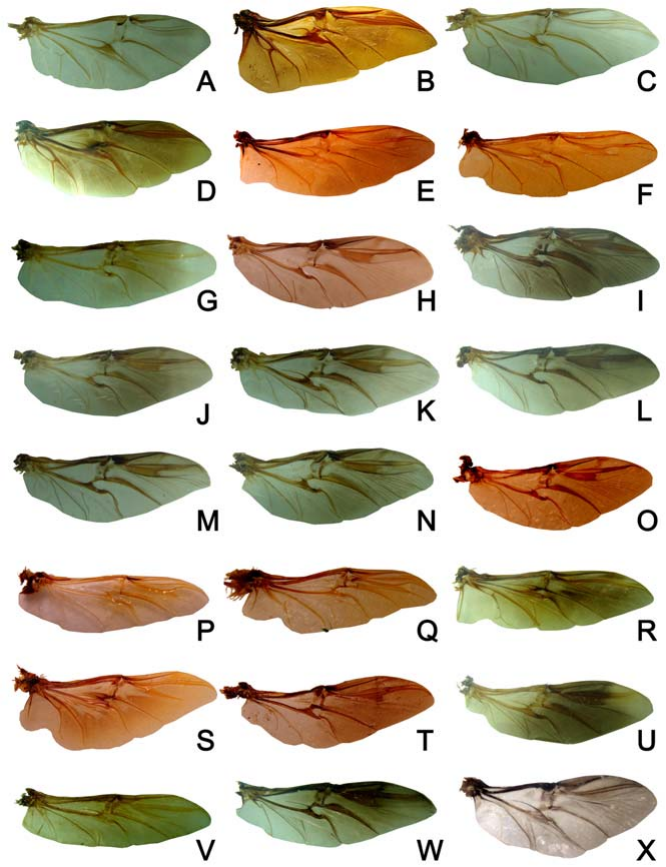
Hind wings of Scarabaeinae from China. (A) *Anoctus laevis*. (B) *Heliocopris dominus*. (C) *Microcopris apicepunctatus*. (D) *Synapsis yunnanus*. (E) *Garreta morosus*. (F) *Paragymnopleurus sinuatus*. (G) *Euoniticellus pallipe*. (H) *Caccobius (Caccophilus) himalayanus*. (I) *Onthophagus (Macronthophagus) diabolicus*. (J) *O. (Palaeonthophagus) gibbulus*. (K) *O. (Parascatonomus) discedens*. (L) *O. (Phanaeomorphus) sycophanta*. (M) *O. (Serrophorus) rectecornutus*. (N) *O. (Strandius) lenzii*. (O) *O. (Altonthophagus) tibetanus*. (P) *Chironitis pamphlius*. (Q) *Onitis philemon*. (R) *Scarabaeus (Kheper) devotus*. (S) *S. (Scarabaeus) sacer*. (T) *Sisyphus (Sisyphus) schaefferi*. (U) *Sinodrepanus rex*. (V) *Oniticellus rahadmistus*. (W) *Liatongus gagatinus*. (X) *Cassolus nudus*.

Nine tribes (100% of Chinese Scarabaeinae tribes, 75.0% of world Scarabaeinae tribes), 26 genera (86.7% of all 30 Chinese Scarabaeinae genera, 11.0% of all 235 world Scarabaeinae genera), and 80 Scarabaeinae species (23.2% of all 345 Chinese Scarabaeinae species, 1.4% of all ∼5700 world Scarabaeinae species) are included in the geometric morphometric and phylogenetic analyses (Figure 9, Table s1). Twelve tribes (100% of world Scarabaeinae tribes), 50 genera (21.3% of all 235 world Scarabaeinae genera), and 50 Scarabaeinae species (0.9% of all ∼5700 world Scarabaeinae species) were included in the phylogenetic analyses of Philips *et al*. (2004). The other four Chinese Scarabaeinae genera (*Cleptocaccobius*, *Haroldius*, *Ochicanthon*, and *Onychothecus*) were not included in the analysis because these genera are rare. One or two species from every genus or subgenus was selected for the analyses. *Aphodius denticulatus* from Aphodiinae was chosen as the outgroup in phylogenetic and morphometric analyses because Aphodiinae is considered the sister taxa to Scarabaeinae [23], [53].

The 119 morphological characters (Table s2) used for analyses and coded for the Chinese dung beetles for the first time were selected from the 200 characters coded in Philips *et al*. [25]. The number of characters was reduced to 119, either because of difficulty in defining or coding discrete states in Chinese species, or because they were autapomorphic and therefore uninformative in Chinese species. We did not exclude any character based on presumptions of possible or probable convergence. Given the large number of characters and taxa used, we hope to propose a more accurate and objective hypothesis of evolutionary history. Detailed illustration for the character states are given elsewhere [25]. The character list and the relative matrix used in the correlation analyses between body features and hind wings are included as table S3. To clarify the role of the hindwing in the evolution of the Scarabaeinae, additional phylogenetic analyses were proposed when both wing and body characters were included (119 characters) and when only body features were included (106 characters).

This study used WinClada software in NONA 2.0 [37], [54] to perform heuristic searches to find the most parsimonious trees. Support for each tree was calculated through bootstrap analysis based on 1000 replications.

Morphometric analysis of variation in dung beetle hind wings was based on 19 landmarks. The 19 landmarks described variation in the entire hind wing morphology. This is the first study to use such a large number of traits to analyze Coleoptera hind wing morphology. The photograph of the outgroup wing (*Aphodius denticulatus*) was taken from Kukalová-Peck & Lawrence [24]. All other 80 wings were photographed with a Sony T9 camera. Cartesian coordinates of the wing landmarks were digitized with tps-DIG 2.05 [55], and landmark configurations were scaled, translated, and rotated against the consensus configuration using the GLS Procrustes superimposition method [56]. The landmark data were analyzed using tps-SMALL 1.2 [38] to compare the distribution of points in the tangent space with their distribution in shape space. The coordinates were analyzed using tps-RELW 1.44 [40] to calculate eigenvalues for each principal warp (Figure 7). Procrustes distances between each of the species were computed using tps-SPLIN 1.20 [57], and the Procrustes distance matrix was analyzed using the unweighted pair group method using arithmetic averages (UPGMA) in NTSYS-pc [58] to determine the phenetic relationships among species (Figure 4A). The Procrustes distances are considered the best method for measuring shape differences among taxa [59], [60], [61], [62], [63], [64].

The average landmark configurations for each tribe were computed using tps-SUPER 1.14 [65] using generalized orthogonal least-squares procedures. The average landmark configurations of the ten taxa (nine tribes and outgroup) were used in the disparity analyses. Disparity is a measure of the amount of morphological variation in a group of samples, which takes into account the volume of the hyper-dimensional morphospace occupied, the relative distances between samples, and the number of samples included in the analysis. The metric disparity score was computed from a partial warps scores matrix, in which the partial warps scores were computed relative to the total mean. The disparity of each tribe was then estimated using COV software (version 102) [66], following the methods of Zelditch *et al*. [67]. Standard errors were generated from 10,000 bootstrap pseudoreplicates.

Landmark data was entered into Mesquite 2.72 [41] as a continuous matrix. The ancestral forms were reconstructed using the landmark drawings module of the Rhetenor package by linking the landmark data and the only tree resulted from NONA in Mesquite.

The matrix correspondence was analyzed using a two-way Mantel test [68], [69] in NTSYS-pc. The correlation analyses of hind wing and body morphology are based on the morphometric data and the sub-matrices of the 119-character matrix, which are indicated with different colors in Table s2 and s3.

## Supporting Information

Table S1 **List of species examined for geometric morphometric and cladistic analyses.**
(DOC)

Table S2 **Morphological characters and their states (following Philips et al., 2004, renumbered).**
(DOC)

Table S3 **Character state matrix for 81 dung beetles species.**
(DOC)

Table S4 **Metric disparity of tribes based on landmark data.**
(DOC)

Table S5 **Correlation analyses of 36 most parsimonious trees and morphometric tree.**
(DOC)

Figure S1 **Head and pronotum (** ***Synapsis*** **).**
(TIF)

Figure S2 **Epipharynx (** ***Heliocopris dominus*** **Bates, 1868).** (A) Dorsal view. (B) Ventral view. (a) Apical margin of distal epipharynx; (b) Fringe; (c) Clypeal-labral suture; (d) Cavity on dorsal side; (e) Median tormal process; (f) Lateral tormal process; (g) Anterior median ventral process; (h) Lateral combs; (i) Closed circles.
(TIF)

Figure S3 **Maxilla (** ***Heliocopris dominus*** **Bates, 1868).** (A) Ventral view. (B) Dorsal view. (a) Maxillary palp; (b) Galea; (c) Sclerite of the galea; (d) Parastipes; (e) Lacinia; (f) Dististipes; (g) Basistipes; (h) Maxacoria; (i) Lateral sclerite; (j) Cardo; (k) Lacinial articulation sclerite.
(TIF)

Figure S4 **Labium (** ***Heliocopris dominus*** **Bates, 1868).** (A) Labial palpus. (B) Paraglossae. (C) Glossae. (D) Distal transverse bridge. (E) Proximal transverse bridge. (F) Apodemes. (G) Mentum. (H) Submentum. (I) Gula. (J) Palpomere strut. (K) Paraglossal strut.
(TIF)

Figure S5 **Mandibles (** ***Heliocopris dominus*** **Bates, 1868).** (A) Right mandible ventral view. (B) Right mandible dorsal view. (a) Incisor; (b) Prostheca; (c) Molar lobe; (d) Apodemes; (e) Incisor lobe; (f) Longitudinal carina; (g) Conjunctivus; (h) Receptacle.
(TIF)

Figure S6 **Metendosternite, dorsal view.** (A) *Heliocopris dominus* Bates, 1868. (B) *Eurysternus*. (C) *Dichotomius*. (D) *Glyphoderus*. (E) *Garreta*. (F) *Anachalcos*. (G) *Cyptochirus*. (H) *Garreta*. (I) *Tragiscus*. (J) *Canthon*. (K) *Copris*. (L) *Kheper*. (M) *Gymnopleurus*. (N) *Epirinus*. (O) *Sulcophanaeus*. (P) *Scaptocnemis*. (Q) *Glyphoderus*. (R) *Anachalcos*. (S) *Kheper*. (T) *Garreta*. (B–T from Philips *et al.*, 2004). (a) Frontal triangle; (b) Furcal arm; (c) Main body; (d) End of main body; (e) Lateral chitinous line in furcal arms.
(TIF)

Figure S7 **Metendosternite, lateral view.** (A) *Heliocopris dominus* Bates, 1868. (B) *Tragiscus*. (C) *Circellium*. (D) *Eurysternus*. (E) *Phanaeus*. (F) *Glyphoderus*. (G) *Kheper*. (H) *Anachalcos*. (I) *Anomiopsoides*. (J) *Garreta*. (K) *Canthon*. (L) *Anachalcos*. (M) *Eurysternus*. (N) *Onthophagus*. (O) *Garreta*. (B–O from Philips *et al.*, 2004). (a) Furcal arm; (b) Frontal midline; (c) Posterior attachment; (d) Midline; (d) Frontal triangle; (f) Lateral chitinous projection.
(TIF)

Figure S8 **Aedeagus.** (A–B) *Liatongus bucerus*. (C–D) *Onthophagus* (*Macronthophagus*) *diabolicus*. (E–F) *Euonthophagus amyntas*. (G–I) *Copris szechouanicus*. (J–L) *Heliocopris bucephalus*. (M–O) *Scarabaeus babori*. (Ventral view: G, J, M; Dorsal view: A, C, E, H, K, N; Lateral view: B, D, F, I, L, O.).
(TIF)

Figure S9 **First tree of the two most parsimonious trees computed from NONA based on 106 characters (wing characters excluded).** Black circles indicate nonhomoplasious changes, white circles indicate changes in homoplasious characters. Number above branches indicate character numbers, below branches indicate character states. Tree length = 854 steps, CI = 0.20, RI = 0.72.
(TIF)

Figure S10 **Strict consensus of the two most parsimonious trees computed from NONA.** Numbers below branches indicate 1000 bootstrap support values. Tree length = 884 steps, CI = 0.19, RI = 0.71.
(TIF)

Figure S11 **Correlation analysis of morphometric tree and first of the two most parsimonious phylogenetic trees.** Matrix correlation: r = 0.53999, significantly correlated at the 1% level.
(TIF)
