# Supplementary material for: Evolutionary Constraints in Hind Wing Shape in Chinese Dung Beetles (Coleoptera: Scarabaeinae)
Source: PLoS One. 2011 Jun 27;6(6):e21600. doi: 10.1371/journal.pone.0021600 (PMC3124545; doi:10.1371/journal.pone.0021600)
Supplement: Table S1 — List of species examined for geometric morphometric and cladistic analyses. (DOC) [file pone.0021600.s001.doc]

## Table s1. List of species examined for geometric morphometric and cladistic analyses

| **Subfamily** | **Tribe** | **Species** |
| --- | --- | --- |
| **Aphodiinae** | Aphodiini | *Aphodius denticulatus* (outgroup) |
| **Scarabaeinae** | Ateuchini | *Parachorius globosus* |
| **Scarabaeinae** | Ateuchini | *Parachorius thomsoni* |
| **Scarabaeinae** | Canthoini | *Cassolus humeralis* |
| **Scarabaeinae** | Canthoini | *Cassolus nudus* |
| **Scarabaeinae** | Canthoini | *Panelus assamensis* |
| **Scarabaeinae** | Canthoini | *Panelus parvulus* |
| **Scarabaeinae** | Coprini | *Catharsius granulatus* |
| **Scarabaeinae** | Coprini | *Catharsius molossus* |
| **Scarabaeinae** | Coprini | *Copris hispanus* |
| **Scarabaeinae** | Coprini | *Copris lunaris* |
| **Scarabaeinae** | Coprini | *Heliocopris bucephalus* |
| **Scarabaeinae** | Coprini | *Heliocopris dominus* |
| **Scarabaeinae** | Coprini | *Microcopris apicepunctatus* |
| **Scarabaeinae** | Coprini | *Paracopris punctulatus* |
| **Scarabaeinae** | Coprini | *Synapsis brahminus* |
| **Scarabaeinae** | Coprini | *Synapsis yunnanus* |
| **Scarabaeinae** | Gymnopleurini | *Garreta morosus* |
| **Scarabaeinae** | Gymnopleurini | *Garreta mundus* |
| **Scarabaeinae** | Gymnopleurini | *Gymnopleurus aciculatus* |
| **Scarabaeinae** | Gymnopleurini | *Gymnopleurus flagellatus* |
| **Scarabaeinae** | Gymnopleurini | *Paragymnopleurus melanarius* |
| **Scarabaeinae** | Gymnopleurini | *Paragymnopleurus sinuatus* |
| **Scarabaeinae** | Oniticellini | *Drepanocerus sinicus* |
| **Scarabaeinae** | Oniticellini | *Euoniticellus fulvus* |
| **Scarabaeinae** | Oniticellini | *Euoniticellus pallipe* |
| **Scarabaeinae** | Oniticellini | *Liatongus gagatinus* |
| **Scarabaeinae** | Oniticellini | *Liatongus phanaeoides* |
| **Scarabaeinae** | Oniticellini | *Oniticellus cinctus* |
| **Scarabaeinae** | Oniticellini | *Oniticellus rahadmistus* |
| **Scarabaeinae** | Oniticellini | *Drepanocerus runicus* |
| **Scarabaeinae** | Oniticellini | *Sinodrepanus rex* |
| **Scarabaeinae** | Onthophagini | *Anoctus laevis* |
| **Scarabaeinae** | Onthophagini | *Anoctus myrmecophilus* |
| **Scarabaeinae** | Onthophagini | *Caccobius (Caccobius) denticollis* |
| **Scarabaeinae** | Onthophagini | *Caccobius (Caccobius) jessoensis* |
| **Scarabaeinae** | Onthophagini | *Caccobius (Caccophilus) himalayanus* |
| **Scarabaeinae** | Onthophagini | *Digitonthophagus gazella* |
| **Scarabaeinae** | Onthophagini | *Euonthophagus amyntas* |
| **Scarabaeinae** | Onthophagini | *Euonthophagus gibbosus* |
| **Scarabaeinae** | Onthophagini | *Onthophagus (Colobonthophagus) armatus* |
| **Scarabaeinae** | Onthophagini | *Onthophagus (Colobonthophagus) tragus* |
| **Scarabaeinae** | Onthophagini | *Onthophagus (Furconthophagus) dapcauensis* |
| **Scarabaeinae** | Onthophagini | *Onthophagus (Gibbonthophagus) atripennis* |
| **Scarabaeinae** | Onthophagini | *Onthophagus (Gibbonthophagus) luridipennis* |
| **Scarabaeinae** | Onthophagini | *Onthophagus (Macronthophagus) diabolicus* |
| **Scarabaeinae** | Onthophagini | *Onthophagus (Macronthophagus) manipurensis* |
| **Scarabaeinae** | Onthophagini | *Onthophagus (Matashia) gracilipes* |
| **Scarabaeinae** | Onthophagini | *Onthophagus (Matashia) kuluensis* |
| **Scarabaeinae** | Onthophagini | *Onthophagus (Micronthophagus) hystrix* |
| **Scarabaeinae** | Onthophagini | *Onthophagus (Micronthophagus) vigilans* |
| **Scarabaeinae** | Onthophagini | *Onthophagus (Onthophagiellus) crassicollis* |
| **Scarabaeinae** | Onthophagini | *Onthophagus (Onthophagus) bivertex* |
| **Scarabaeinae** | Onthophagini | *Onthophagus (Onthophagus) taurus* |
| **Scarabaeinae** | Onthophagini | *Onthophagus (Palaeonthophagus) gibbulus* |
| **Scarabaeinae** | Onthophagini | *Onthophagus (Palaeonthophagus) vacca* |
| **Scarabaeinae** | Onthophagini | *Onthophagus (Paraphanaeomorphus) argyropygus* |
| **Scarabaeinae** | Onthophagini | *Onthophagus (Paraphanaeomorphus) trituber* |
| **Scarabaeinae** | Onthophagini | *Onthophagus (Parascatonomus) discedens* |
| **Scarabaeinae** | Onthophagini | *Onthophagus (Parascatonomus) funebris* |
| **Scarabaeinae** | Onthophagini | *Onthophagus (Phanaeomorphus) fodiens* |
| **Scarabaeinae** | Onthophagini | *Onthophagus (Phanaeomorphus) sycophanta* |
| **Scarabaeinae** | Onthophagini | *Onthophagus (Proagoderus) amplexus* |
| **Scarabaeinae** | Onthophagini | *Onthophagus (Proagoderus) yunnanus* |
| **Scarabaeinae** | Onthophagini | *Onthophagus (Serrophorus) rectecornutus* |
| **Scarabaeinae** | Onthophagini | *Onthophagus (Serrophorus) seniculus* |
| **Scarabaeinae** | Onthophagini | *Onthophagus (Strandius) janonicus* |
| **Scarabaeinae** | Onthophagini | *Onthophagus (Strandius) lenzii* |
| **Scarabaeinae** | Onthophagini | *Onthophagus (Sunenaga) anguliceps* |
| **Scarabaeinae** | Onthophagini | *Onthophagus (Altonthophagus) cupreiceps* |
| **Scarabaeinae** | Onthophagini | *Onthophagus (Altonthophagus) tibetanus* |
| **Scarabaeinae** | Onitini | *Chironitis arrowi* |
| **Scarabaeinae** | Onitini | *Chironitis pamphlius* |
| **Scarabaeinae** | Onitini | *Onitis philemon* |
| **Scarabaeinae** | Scarabaeini | *Scarabaeus (Kheper) devotus* |
| **Scarabaeinae** | Scarabaeini | *Scarabaeus (Kheper) erichsoni* |
| **Scarabaeinae** | Scarabaeini | *Scarabaeus (Scarabaeus) sacer* |
| **Scarabaeinae** | Scarabaeini | *Scarabaeus (Scarabaeus) typhon* |
| **Scarabaeinae** | Sisyphini | *Sisyphus (Neosisyphus) bowringi* |
| **Scarabaeinae** | Sisyphini | *Sisyphus (Neosisyphus) spinipes* |
| **Scarabaeinae** | Sisyphini | *Sisyphus (Sisyphus) schaefferi* |
