# Supplementary material for: Evolutionary Constraints in Hind Wing Shape in Chinese Dung Beetles (Coleoptera: Scarabaeinae)
Source: PLoS One. 2011 Jun 27;6(6):e21600. doi: 10.1371/journal.pone.0021600 (PMC3124545; doi:10.1371/journal.pone.0021600)
Supplement: Table S2 — Morphological characters and their states (following Philips et al., 2004, renumbered). (DOC) [file pone.0021600.s002.doc]

## Table s2. Morphological characters and their states (following Philips et al., 2004, renumbered)

| **Head (Figure s1)**   1. Clypeal teeth number and development: (0) none; (1) two poorly developed; (2) two distinct, well developed; (3) three; (4) four distinct; (5) four with two lateral poorly developed. 2. Genal development laterally: (0) poor to slight; (1) moderate to great. 3. Genal lateral edge: (0) approximately straight to slightly (obtusely) angled; (1) acutely angled. 4. Gula apical 1/2 medially: (0) lacking a triangular band of setae; (1) with a triangular band of fine setae; (2) with an extremely elongate band of fine setae. 5. Gula lateral edge: (0) with a distinct band of fine setae; (1) without a distinct band of fine setae. 6. Gula proximal margin: (0) pointed; (1) truncate or slightly emarginate; (2) distinctly emarginate. 7. Frons: (0) without any type of projection; (1) with a transverse ridge; (2) with a median horn or protuberance, of various lengths. 8. Eye canthus: (0) completely dividing eye into upper and lower halves; (1) or not. |
| --- |
| **Antennae**   1. Seventh antennomere: (0) relatively elongate and parallel sided; (1) relatively curved and cup-shaped or rounded. 2. Apical antennomere: (0) about as long as scape or slightly longer (excluding the basal insertion point or ‘ball’); (1) distinctly shorter. 3. Seventh and eighth antennomeres: (0) small cavity on anterior surface; (1) cavity absent.   **Epipharynx (Figure s2)**   1. Lateral combs: (0) without any distinct rows; (1) single row; (2) double row, both of single setal lines; (3) double row with the second row composed of a band of setae. 2. Anterior median ventral process: (0) broad throughout entire length; (1) or not. 3. Lateral tormal process proximally: (0) without closed circles and no ‘hooks’; (1) with closed circles (although sometimes indistinct); (2) without closed circles and with ‘hooks’; (3) with only one closed circle. 4. Apical margin of distal epipharynx: (0) darkened/pigmented; (1) or not. 5. Apical margin of distal epipharynx, excluding anterior process if projecting: (0) narrowly indented at middle; (1) broadly shallowly indented; (2) approximately truncate; (3) deeply indented; (4) broadly rounded; (5) sinuate, with a lateral and medial indentation or semi-sinuate, with margin transverse laterally; (6) slightly pointed; (7) with three anterior projections, the median shortest; (8) with one small but distinct median projection in a shallow emargination; (9) rounded but with a shallow emargination. 6. Proximal epipharynx near transverse median suture (‘clypeal-labral’ suture): (0) laterally acutely lobed anteriorly; (1) laterally straight or rounded. 7. Apical margin of distal epipharynx: (0) setal fringe long, > 1/2 length of distal epipharynx; (1) fringe short, < 1/2 length of distal epipharynx. 8. Cavity on dorsal side of proximal portion of epipharynx: (0) reaching clypeal-labral suture; (1) not reaching suture; (2) cavity absent. 9. Ventral surface: (0) with distinctly larger coarse scattered setae among the smaller setae; (1) or large setae absent. 10. Lateral tormal arms: (0) diverging posteriorly; (1) parallel or converging slightly posteriorly.   **Maxilla (Figure s3)**   1. Apex of ventral articulatory sclerite of the galea (distal half) in ventral view: (0) tapered and coming to a point; (1) transversely blunt or broadly rounded. 2. Galeal shape: (0) approximately transverse; (1) or relatively ovoid. 3. Proximal inner strut of lacinia (nearest to palpifer): (0) distal tip extending out from lacinial body as a parallel sided finger; (1) or not. 4. Apical palpomere: (0) apical 1/5 - 2/5 distinctly lighter than base (although sometimes a gradual transition zone at apical 2/5); (1) or not. 5. Distal half of ventral articulatory sclerite (‘V’) of the galea: (0) expanded and thickened (recurved) towards tip; (1) approximately parallel sided for most of its length. 6. Distal half of ventral articulatory sclerite (‘V’) of the galea at apex: (0) approximately straight; (1) curved into a distinct hook. 7. Lateral sclerite notch where palp inserts (viewed with sclerite flat): (0) sides on either side of notch distinctly asymmetrical; (1) or not. 8. Outer tooth of lacinial articulation sclerite (tooth angled away from lacinia onto adjacent sclerite): (0) pointing anteriorly or nearly so; (1) pointing laterally; (2) tooth absent. 9. Ventral articulatory sclerite in dorsal view of the galea: (0) apex of distal portion recurved; (1) or not. 10. Ventral articulatory sclerite (‘V’) anterior-laterally: (0) with an extra acute projection; (1) a rounded projection; (2) without a projection (straight or tapered but may be curved); (3) or with a small tooth.   **Labium (Figure s4)**   1. Number of palpomeres: (0) two; (1) three. The basal piece is part of a larger sclerite, the palpomere strut, and is not being counted as a palpomere. 2. Second palpomere width compared to the first: (0) about the same width or slightly wider; (1) obviously narrower. 3. Shape of the first palpomere: (0) cylindrical or nearly so; (1) subtriangular; (2) triangular but without a basal stalk; (3) triangular and with a basal stalk. 4. Mentum apical edge medially: (0) rounded; (1) shallow V-shaped notch; (2) deep V-shaped notch; (3) U-shaped notch. 5. Mentum apical margin near lateral edge: (0) distinctly notched to slightly concave; (1) round or straight. 6. Glossal internal strut (in lateral view): (0) narrow, approximately parallel sided, not expanded apically; (1) gradually expanded apically; (2) abruptly expanded apically; (3) broad throughout most of its length. This internal strut contacts the proximal end of the palpomere strut. The palpomere strut has its distal end located near or at the base of the first palpomere. 7. Mentum: (0) distinctly transverse; (1) length about equal to width. 8. Mentum lateral edge posterior of middle: (0) straight to slightly or distinctly rounded; (1) slightly sinuate with a shallow notch. 9. Paraglossae: (0) bifurcate (i.e. with inner and outer lobe); (1) or not. 10. Paraglossal apex (outer apex, if bilobed): (0) triangular or rounded lobe; (1) truncate; (2) rounded and medially acute. 11. Paraglossal strut in lateral view: (0) extending past apex of recurved portion of palp strut (apodeme); (1) or ending/ meeting at apex. 12. Paraglossal strut at proximal apex: (0) distinctly bifurcate; (1) or not. 13. Palpomere strut on recurved portion in lateral view: (0) with a tooth-shaped lobe facing posteriorly; (1) lobe absent. This lobe is located near the end of the strut close to where the paraglossal strut is located. 14. Paraglossal lobes: (0) diverging and usually distinctly widely separated; (1) approximately parallel or slightly converging and usually closely spaced. 15. Shape of the anterior declivous region of the mentum: (0) broad and elongate; (1) narrow and elongate; (2) broad and short; (3) region lacking, at most a small lip; (4) broad and elongate but a separate sclerite. 16. Glossae (between base of paraglossae): (0) two distinct anteriorly directed lobes (with a median emargination); (1) one small median lobe; (2) two elongate and distinct lobes (although medially fused for most of their length; (3) lobes apparently lacking. 17. Most proximal apodemes (oval or round muscle attachment points attached to stalks) at their apex: (0) with radiating darkened lines; (1) lines absent. 18. Apodeme (V- or Y-shaped) adjacent to the paraglossal apodeme in dorsal view: (0) V-shaped; (1) Y-shaped ‘down’ (branch from about middle or beyond); (2) Y-shaped ‘up’; (3) straight. These apodemes are located posterior of the paraglossal lobes. 19. Posterior arm (excluding proximal apex) of V- or Y-shaped apodeme adjacent to the paraglossal apodeme in dorsal view, anterior to posterior: (0) distinctly angled obliquely inward; (1) approximately parallel; (2) distinctly angled obliquely outward; (3) parallel and then converging; (4) parallel and then diverging. 20. Proximal transverse bridge of the posterior labial struts (shape of the dark and light coloured portion): (0) a broad triangular or ear shaped lobe; (1) approximately an ovoid lobe; (2) a flat pad; (3) somewhat amorphous. 21. Longitudinal arms adjacent to distal transverse bridge in ventral view: (0) with narrow parallel sided arms extending out laterally from triangular base; (1) with triangular base only (and sometimes just a small ‘tooth’); (2) with broad parallel sided arms extending laterally; (3) arms apparently lacking. 22. Distal transverse bridge in ventral view; proximal edge, excluding thinner sclerotised cuticle: (0) evenly rounded and/or approximately transverse; (1) distinctly oblique or with sides uneven; (2) transverse with two notches on each side. 23. Dorso-ventral arm from distal bridge in ventral view: (0) arising medially; (1) arising on side.   **Mandibles: ventral view (Figure s5A)**   1. Apex of sclerotised area near inner lateral margin: (0) with a distinct channel ending in an enlarged ball-shaped tip; (1) or absent. 2. Sclerotised area between longitudinal carina and inner lateral edge, with sensilla: (0) present as one fairly linear, widely spaced row; (1) scattered. This region is visible in the illustrations as an irregular patch or a line of dots representing the sensilla. 3. Sclerotised area between longitudinal carina and inner lateral edge: (0) long setae present; (1) absent. 4. Distinct longitudinal carina near lateral edge at middle (i.e. with a nearly horizontal area lateral to carina visible from above): (0) present; (1) absent. 5. Apical reach of mesal comb compared to sclerotised area: (0) sclerotised area shorter than mesal comb; (1) sclerotised area about the same length as mesal comb; (2) mesal comb absent.   **Mandibles: dorsal view (Figure s5B)**   1. Receptacle shape at proximal margin: (0) approximately rounded; (1) reduced; (2) broadly and obviously truncate; (3) broadly but subtlety truncate; (4) with a narrow projection. 2. Receptacle towards brush/ comb side: (0) distinctly laterally notched; (1) or not. |
| **Pronota and prolegs**   1. Number of teeth on tibia: (0) four; (1) three. 2. Tarsi: (0) present; (1) absent. 3. Tibial apex in males: (0) spine absent (although a spine-like tooth present); (1) with a large socketed spine; (2) with a socketed lobe or paddle. 4. Tibia: (0) with a longitudinal carina on inner dorsal surface (not outer surface adjacent to declivity); (1) carina absent. 5. Femoral setal brush at base: (0) with only long setae; (1) with both long and short setae in two separate, but juxtaposed patches. 6. Pronotum lateral edge: (0) smooth; (1) serrated. 7. Pronotal surface: (0) smooth, unsculptured, smoothly convex; (1) protuberance or one or more cavities present. 8. Pronotum: (0) with a small dimple or depression present at basal margin; (1) without small dimple or depression at basal margin. 9. Protibia between second and third teeth: (0) microteeth absent; (1) one or more microteeth present.   **Meso- and metasterna, including legs**   1. Metasternum posteriorly: (0) with both a depression at middle and at sides; (1) with a depression on either side of middle; (2) with a wide depression at middle; (3) depression absent. 2. Metasternum: (0) with an anteriorly directed acutely pointed ridge or spine; (1) or not. 3. Mesosternum of male, viewed laterally: (0) with a posteriorly directed, slightly projecting edge; (1) or not. 4. Metatarsal claws: (0) present; (1) absent. 5. Mesotibial apex: (0) with two large spines; (1) with one large spine; (2) spines absent. 6. Metatarsi: (0) first tarsomere distinctly longer than fifth; (1) first distinctly shorter than fifth; (2) first about equal to fifth. 7. Metatarsi: (0) first metatarsomere distinctly shorter than second; (1) about equal to second; (2) slightly longer than second (1.1–1.5); (3) greatly longer than second (~2 or more). 8. Orientation of midcoxae: (0) strongly oblique (~45°); (1) slightly oblique (~10–25°); (2) approximately parallel; (3) approximately parallel and then strongly oblique anteriorly. 9. Metatibial expansion towards apex: (0) absent or slight (sometimes expanded just at apex); (1) moderately expanded (approximately parallel on basal half); (2) greatly expanded throughout; 10. Metatibial apex: (0) one spine; (1) two spines |
| **Elytra**   1. Epipleura: (0) notch present; (1) notch absent. 2. Elytral surface: (0) finely tuberculate; (1) without tuberculate.   **Hindwing**   1. Length of MP: (0) long; (1) short; (2) absent. 2. Pigmentation near apex: (0) no distinct patch; (1) distinct and truncate to slightly emarginate distally. Taxa with a very slight amount of pigmentation were coded as 0. 3. Shape of RA: (0) abruptly curved anteriorly near margin; (1) straight through to margin (but curving posteriorly at margin); (2) distinctly sigmoidal. 4. Amount of long setae along costal margin (flying hairs): (0) moderate amount, from costal vein break up to half the distance towards base; (1) large amount, extending past midway point to near base. 5. Jugal vein: (0) present (may be quite faint); (1) absent, at most a short stub. Species have either a distinct jugal vein or it is completely absent or at most a barely perceptible stub proximally 6. AA vein: (0) with a distinct branch or tooth near base forming or nearly forming an elongate cell with distal posterior angle about or greater than 90º; (1) cell absent; (2) with vein forming an elongate cell with distal posterior angle obviously less than 90º. 7. RP-RA veins: (0) distinctly visible as two separate, approximately parallel veins; (1) visible as a ‘single’ vein since the two veins are juxtaposed (although sometimes slightly separated over part of their length and only fused apically); (2) distinctly visible as two separate, but not parallel, veins. 8. AA vein shape: (0) bifurcate near posterior margin; (1) or not. 9. Setae along costal margin: (0) a separate patch near wing base; (1) near wing base without separate patch. 10. Anal region: (0) ‘normal’ with a distinct lobe; (1) reduced, lacking any distinct lobe. 11. Costal margin at wing base: (0) with fine striae; (1) or not. |
| **Metendosternite, dorsal view (Figure s6)**   1. Distal position of lateral chitinous line in furcal arms: (0) anterior; (1) middle; (2) posterior. 2. Shape of lateral chitinous line in furcal arms: (0) straight; (1) slightly concave anteriorly; (2) very concave anteriorly. 3. Posterior form of furcal arms: (0) convex; (1) concave; (2) straight; (3) straight then convex; (4) curved. 4. Direction of furcal arm tip: (0) projected posteriorly to the back; (1) projected laterally; (2) projected anteriorly to the front. 5. Furcal tip shape apically: (0) bluntly rounded; (1) acute lobe projecting anteriorly; (2) acute lobe projecting posteriorly; (3) bilobed; (4) tri-lobed. 6. Width of furcal arm: (0) approximately parallel sided; (1) increasing in width; (2) narrowing in width. 7. Chitinous line just posterior of furcal arms: (0) continuous through to sides; (1) with a break at middle; (2) present only at middle, between two chitinous lines; (3) line absent. 8. Form of midline: (0) dorsal projection present; (1) absent (i.e. completely flat). 9. Shape of main body towards posterior end: (0) similar width throughout; (1) narrowing; (2) widening.   **Metendosternite, lateral view (Figure s7)**   1. Frontal triangle, projection directed: (0) anteriorly; (1) ventrally; (2) posteriorly. 2. Frontal triangle position relative to the remaining metendosternite: (0) lower; (1) aligned; (2) higher. 3. Frontal tip shape: (0) rounded; (1) wide triangle; (2) narrow triangle; (3) right angled. 4. Orientation of frontal midline: (0) straight throughout; (1) abruptly rounded; (2) gradually rounded. 5. Frontal midline position at apex: (0) not parallel; (1) parallel only at apex; (2) parallel well before apex. 6. Shape of lateral chitinous projection: (0) erect throughout length; (1) erect only partially; (2) flat. 7. Width near posterior attachment: (0) narrow; (1) moderate; (2) wide. |
| **Ventrites**   1. First ventrite: (0) distinctly visible throughout entire length; (1) reduced to a thin line near middle and barely discernable. 2. First ventrite, visible portion, anterior projection between metacoxae: (0) blunt broadly rounded lobe; (1) truncate (various widths); (2) pointed. 3. Third ventrite at middle compared to lateral edge: (0) reduced slightly, down to ~1/2 the length of that at lateral edge; (1) reduced greatly, only 2/5 or less the length at lateral edge. 4. Pseudoepipleura (in lateral view): (0) absent; (1) with pseudoepipleura; (2) with double pseudoepipleura.   **Pygidium**   1. Anterio-medial groove: (0) distinct with complete lateral edges throughout; (1) indistinct or faintly visible; (2) completely absent. 2. Transverse ridge: (0) strong and distinct, often with a slight ventrally directed overhanging edge; (1) slightly pronounced; (2) absent. 3. Shape of dorso-lateral edge: (0) rounded; (1) angled; (2) ‘squared off ’ with flattened edges; (3) knobbed or distinctly pointed. 4. Dark band of pigment at base of the propygidium: (0) present; (1) absent. |
| **Aedeagus (Figure s8)**   1. Parameres in lateral view: (0) parameres distinctly tapered to a point; (1) somewhat blunt or truncate, although an acute apex may be present. 2. Paramere position relative to basal piece: (0) perpendicular; (1) obliquely angled; (2) acutely angled. |
