# Supplementary material for: Evolutionary Constraints in Hind Wing Shape in Chinese Dung Beetles (Coleoptera: Scarabaeinae)
Source: PLoS One. 2011 Jun 27;6(6):e21600. doi: 10.1371/journal.pone.0021600 (PMC3124545; doi:10.1371/journal.pone.0021600)
Supplement: Table S3 — Character state matrix for 81 dung beetles species. (DOC) [file pone.0021600.s003.doc]

## Table s3. Character state matrix for 81 dung beetles species

| Characters  Taxa | 00000000  00000000  01234567 | 00000000000000000000000000000000000000000000000000000  001111111111122222222223333333333444444444455555555556  89012345678901234567890123456789012345678901234567890 | 0000000000000000000  6666666666777777777  1234567890123456789 | 0000000000000  8888888888999  0123456789012 | 00000001111111111  9999999000000000  3456789012345678 | 111111111  011111111  90123456 | 11  11  78 |
| --- | --- | --- | --- | --- | --- | --- | --- |
| ***Aphodius denticulatus* (outgroup)**  ***Parachorius globosus***  ***Parachorius thomsoni***  ***Cassolus humeralis***  ***Cassolus nudus***  ***Panelus assamensis***  ***Panelus parvulus***  ***Catharsius granulatus***  ***Catharsius molossus***  ***Copris hispanus***  ***Copris lunaris***  ***Heliocopris bucephalus***  ***Heliocopris dominus***  ***Microcopris apicepunctatus***  ***Paracopris punctulatus***  ***Synapsis brahminus***  ***Synapsis yunnanus***  ***Garreta morosus***  ***Garreta mundus***  ***Gymnopleurus aciculatus***  ***Gymnopleurus flagellatus***  ***Paragymnopleurus melanarius***  ***Paragymnopleurus sinuatus***  ***Drepanocerus sinicus***  ***Euoniticellus fulvus***  ***Euoniticellus pallipe***  ***Liatongus gagatinus***  ***Liatongus phanaeoides***  ***Oniticellus cinctus***  ***Oniticellus rahadmistus***  ***Drepanocerus runicus***  ***Sinodrepanus rex***  ***Anoctus laevis***  ***Anoctus myrmecophilus***  ***Caccobius (Caccobius) denticollis***  ***Caccobius (Caccobius) jessoensis***  ***Caccobius (Caccophilus) himalayanus***  ***Digitonthophagus gazella***  ***Euonthophagus amyntas***  ***Euonthophagus gibbosus***  ***Onthophagus (Colobonthophagus) armatus***  ***O. (Colobonthophagus) tragus***  ***O. (Furconthophagus) dapcauensis***  ***O. (Gibbonthophagus) atripennis***  ***O. (Gibbonthophagus) luridipennis***  ***O. (Macronthophagus) diabolicus***  ***O. (Macronthophagus) manipurensis***  ***O. (Matashia) gracilipes***  ***O. (Matashia) kuluensis***  ***O. (Micronthophagus) hystrix***  ***O. (Micronthophagus) vigilans***  ***O. (Onthophagiellus) crassicollis***  ***O. (Onthophagus) bivertex***  ***O. (Onthophagus) taurus***  ***O. (Palaeonthophagus) gibbulus***  ***O. (Palaeonthophagus) vacca***  ***O. (Paraphanaeomorphus) argyropygus***  ***O. (Paraphanaeomorphus) trituber***  ***O. (Parascatonomus) discedens***  ***O. (Parascatonomus) funebris***  ***O. (Phanaeomorphus) fodiens***  ***O. (Phanaeomorphus) sycophanta***  ***O. (Proagoderus) amplexus***  ***O. (Proagoderus) yunnanus***  ***O. (Serrophorus) rectecornutus***  ***O. (Serrophorus) seniculus***  ***O. (Strandius) janonicus***  ***O. (Strandius) lenzii***  ***O. (Sunenaga) anguliceps***  ***O. (Altonthophagus) cupreiceps***  ***O. (Altonthophagus) tibetanus***  ***Chironitis arrowi***  ***Chironitis pamphlius***  ***Onitis philemon***  ***Scarabaeus (Kheper) devotus***  ***Scarabaeus (Kheper) erichsoni***  ***Scarabaeus (Scarabaeus) sacer***  ***Scarabaeus (Scarabaeus) typhon***  ***Sisyphus (Neosisyphus) bowringi***  ***Sisyphus (Neosisyphus) spinipes***  ***Sisyphus (Sisyphus) schaefferi*** | 00001101  10001100  10001100  50001100  50001100  10001100  10001100  01001121  01001121  11101121  11101121  00000111  00000111  10001101  11101121  21100121  21100121  50020201  50020201  10020201  10020201  10020201  10020201  10001001  10001020  10001020  00001100  00001100  10001100  10001100  10001001  10001100  10000110  10000110  10000110  10000110  10000120  00001120  00001110  00001110  00001110  00001110  00001110  00001110  00001110  00001110  00001110  00001110  00001110  00001110  00001110  00001110  00001110  00001110  00001110  00001110  00001110  00001110  30001110  00001110  00001110  00001110  00001110  00001110  00001110  00001110  00001110  00001110  00001110  00001110  00001110  10001110  10001110  00001110  41100100  41100100  41120100  41120100  10001101  10001101  10001101 | 10111012212110110100013101110101011100313200001111001  11010102111000110101002100101101001100110012111011211  11010102111000100101012100101101001100110012111011031  11110002111010110101201100101101001100110012111111120  11110202111000110101201100101101001100110012111111120  10001112010101010011202101101101111100011003001011121  10001112010101010001202101101101111100011003001011121  00111111112001101011001100200100001101003203211001001  00111101112001101011001100200100001100003203211001001  11111118111011110011201100101100011100110301001101120  11111118110011110011101100102100011100110001001101120  01111104101111101110202112301000001111111303210100141  01111104101111101110102112301000001111111303210100141  11111018010011110111002102101100001100011103000101020  11111113110011011111102101200100001110111003110100031  01111112101011111011201111210100001100101003211100100  01111112101011111011201111210100001100101003211100100  11111112110011100010001100112101011100110101111110121  11111112110011100010001100112101011100110101111110121  11111012011011100011102110101101000100111002211101031  11111002011011101011102110110101000111111002211101031  11111111010011101010002111201101010010111113010101130  11111111010011100010002111201101010010111113010101110  11011101101101010110012001100101011100110100111110001  11011111101001011101012112102011011100110200111110001  11011111101001011101012112102011011100110200111110001  11011112001000010101012001102011011100010100111110001  11011112001000010101012001102011011100010100111110001  11011111011001010101012002102111011100310100111110001  11011111011001010101012002102111011100310100111110001  11011101101100010110012001100101011100110100111110001  11011105111000000010202002100001010010101032110011040  11011111111100000101202100110101011110311031001011001  11011111111100000101202102110101011110311031001011001  11011102111100100011200100100001111110311131000011031  11011102111100100011200100100001111110311131000011031  11011106111101100001200100100001111110311131000011031  11011100011011001001001101100101201110111402011011041  11011104111011000001011101100101011000301033000011001  11011102111011000001011101100101011100301033000011001  11011102011011000100102100101111111100110413111011001  11011102011011000000102100101111111100110413111011001  11011102011001010000202100101111011100110403111011001  11011102111111011000202101101111011100110403111011001  11011102111111010000202101101111011100110403111011001  11011111111001100001203102110101001110110401111001000  11011111111001100001203102110101001110110401111001000  11011101011000100001212101100001001100110403111011100  11011102011000000001212101100001001100110403111011100  11011112111001110001201100101101011100110401111011100  11011112111001110001201100101101011100110401111011100  11011101111000011001212101102101011100110401111011100  11011102111000011001212101101101011100110401111011100  11011101111000011001212101101101011100110401111011100  11011102111001000001212101101101011100110401111011000  11011102111001000001212101101101011100110401111011000  11011101111100011101112101101101011100110401111011000  11011101111100011101112101101101011100110401111011000  11011000012100011101112101101101011100110401111011040  11011108012100011101112101101101011100110401111011040  11011101111011110101212100101101011100110403111011000  11011100111011010101212100101101011100110403111011000  11011112112010010000200101101001011100110403011011000  11011112112010010000202101101001011100110403011011000  11011110010111011101201100101001011100110403111011000  11031111011111011101201100101001011100110403111011000  11011102011011010001212102201101011100110403111011000  11011102011011110001212102201101011100110403111011000  11011101111011001101202101101101011100110403111011000  11011102111110000000001100100101001100010403010011101  11011102111110000010001100100101001100010403011011101  11021110111010000111002100100001010100011432011101021  11031310111010000111001100100001010100011431111101021  11131111111010101110002102202001001100011401100101031  11131004111010111010212100010101000102010331111101120  11131004111010111010212100010101000102010331111101120  11111011101010111010212100010101000102000301111101120  11111011101010111010212100010101000102000301111101120  11111311111000100101002111200101011100110002110110021  11111311111000100101002111200101011100110002110110021  11111311111001100101012111200101011100010102110110021 | 1011000103110002321  1001000101100003220  1011000111100003220  1011000113110021000  1011000113110021000  1011000113110010200  1011000113110010200  1011001100010003220  1011001100010003220  0011001102110022220  0011001102110022220  1010001102110002120  1010001102110002120  0011000102110003220  0011000102110003220  1010000112110002210  1010000112110002210  1010000113110112100  1010000113110112100  1010000113110112100  1010000113110112100  1010000113110112100  1010000113110112100  0011001102110003200  0011000103110003220  0011000103110003220  0011001103110003220  0011001103110003220  0010000103110003220  0010000103110003220  0011001102110003200  1010001103110003220  0011001103110003120  0011001103110003120  0011001103110003120  0011000103110003120  0011001103110003120  0011001103110003120  0011001102110003120  0011001101110003120  0011001103110003120  0011001103110003120  0011001103110003120  0011001103110003120  0011001103110003120  0011001103110003120  0011001103110003120  0011001103110003120  0011001103110003120  1011000113110003120  1011000113110003120  0011001103110003120  0011001103110003120  0011001103110003120  0011001103110003120  0011001103110003120  0011001103110003120  0011001103110003120  0011001103110003120  0011001103110003120  0011001113110003120  0011001113110003120  0011001103110003120  0011001103110003120  0011001103110003120  0011001103110003120  0011001113110003120  0011001113110003120  0011001103110003120  0011001103110003120  0011001103110003120  0001010001100003220  0001010001100003220  0100000001100002220  0110110103110212000  0110110103110212000  0110110113110112000  0110110113110112000  1011000112110013200  1011000112110013200  1011000112110023200 | 1100001101101  1100110001011  1100110001011  1100100001011  1100100001011  1100111001111  1100111001111  1100110001001  1100110001001  1100100001001  1100100001001  1100100001001  1100100001001  1100100001101  1100100001001  1100110001101  1100110001101  0000200120111  0000200120111  0000200120111  0000200120111  0000200120111  0000200120111  1101001111011  1111001211111  1111001211111  1101000211110  1101001211110  1100011211110  1100011211110  1101001111011  1101001111111  1111101101111  1111101101111  1111101111110  1111101111110  1111111101111  1101100201111  1111101111111  1111101111111  1121101111110  1121101111110  1121100111110  1121000211110  1121000211110  1110100211110  1110100211110  1121101011110  1121101011110  1111101101110  1111101101110  1111201111110  1121101111110  1121101111110  1111101211110  1111101211110  1111101111110  1111101111110  1101101211110  1101101211110  1110000211110  1110000211110  1101001011110  1101001011110  1121000211110  1121000211110  1111000211110  1111000211110  1111000211110  1111101111110  1101101101110  1100200001111  1100200001111  1100210001011  1000100010011  1000100010011  1100110000011  1100110000011  1121101111111  1121101111111  1121111101111 | 1122002010020002  0130210112232010  1130210112232010  0130210112232020  0130210112232000  1102420012100000  0102420012100000  1200103011110100  1200103011110100  2100022010211000  1110022010210100  2131012011201000  2131012011201000  1000210000201200  1001310100200100  2200000020210100  2200000020210100  0142100020012210  0142100020012210  1121310001011210  1122110000011210  1142120021120100  1142120021120100  0111410120022100  0111311020121010  0111311020121010  0111310010022200  0111310010022200  2140313110022210  2140313110022210  0111410120022100  1241313020220210  2211300000232110  0210200000232110  1231310010201210  1231310010201210  1231310010210100  0201313021201210  1131313000202210  1131313000202210  0121310010212210  1141310000212210  0211310000212110  1241310000222210  1241310010222210  0131312020210210  0131312020210210  0131310010212110  1131310000212110  0121310000112210  0121310000112210  0131310010212110  1131310010212100  1131310010212110  1131310010212110  1131310010212100  0231310010212100  0231310010212100  0241310011212110  0241310011212110  1141310011212210  1141310011212210  1241310120112200  2241310020112200  1241310000212200  1241310020212200  1241310021212200  1241310021212200  1241310010212210  1201310010202210  1201310010202210  1242103020210110  1242123120210110  1222310021021200  2040413010011201  2040413010011201  1130010010011000  1130010010011000  0110211120022000  0110211120022000  0142211020131010 | 00002221  12100011  12100011  02101010  02101010  12010011  12010011  02110010  02110010  12110011  12110011  02110001  02110001  12110011  12110011  02110011  02110011  02101001  02101001  02100001  02100001  02102001  02102001  01010011  01002210  01002210  01002210  01002211  02002210  02002210  01010011  02012010  02102111  02102111  02102111  02102111  02102111  02102020  02102101  02102101  02102121  02102121  02102021  02102121  02102121  02102121  02102121  02102121  02102121  02102121  02102121  02102121  02102121  02102121  02102121  02102121  02102121  02102121  02102121  02102121  02102121  02102121  02102121  02102121  02102121  02102121  02102121  02102121  02102121  02102121  02102121  02100011  02100011  02022011  02121011  02121011  02021011  02021011  01100011  01100011  01100011 | 01  11  11  11  11  00  00  01  01  01  01  01  01  11  11  01  01  01  01  01  01  01  01  11  11  11  11  11  11  11  11  11  00  00  12  12  11  11  10  00  11  10  10  10  10  10  10  10  10  10  10  11  11  11  11  11  11  11  11  11  10  10  11  11  11  11  12  10  11  00  00  11  11  11  01  01  11  11  01  01  01 |
