# Supplementary material for: Evolutionary Constraints in Hind Wing Shape in Chinese Dung Beetles (Coleoptera: Scarabaeinae)
Source: PLoS One. 2011 Jun 27;6(6):e21600. doi: 10.1371/journal.pone.0021600 (PMC3124545; doi:10.1371/journal.pone.0021600)
Supplement: Table S4 — Metric disparity of tribes based on landmark data. (DOC) [file pone.0021600.s004.doc]

## Table s4. Metric disparity of tribes based on landmark data

MD =Metric disparity; SE = standard error.

|  | **Landmarks** | |
| --- | --- | --- |
|  | **MD** | **SE** |
| **AT** | 319210132.0 | 22303573.2 |
| **CA** | 205665217.7 | 4419433.7 |
| **CO** | 184843672.4 | 29260198.2 |
| **GY** | 180155443.8 | 4680226.6 |
| **ON** | 156734397.5 | 5668268.4 |
| **OP** | 145458410.1 | 1458114.1 |
| **OT** | 206859505.5 | 58858534.7 |
| **SC** | 208402602.0 | 5854926.0 |
| **SI** | 211858971.0 | 9920706.8 |
