# Supplementary material for: Evolutionary Constraints in Hind Wing Shape in Chinese Dung Beetles (Coleoptera: Scarabaeinae)
Source: PLoS One. 2011 Jun 27;6(6):e21600. doi: 10.1371/journal.pone.0021600 (PMC3124545; doi:10.1371/journal.pone.0021600)
Supplement: Table S5 — Correlation analyses of 36 most parsimonious trees and morphometric tree. (DOC) [file pone.0021600.s005.doc]

## Table s5. Correlation analyses of most parsimonious trees and morphometric tree

* Out of 3000 random permutations, indicated the numbers which < Z, = Z or > Z (Z: observed Z-value, see Methods parts for details)

|  | **Matrix correlation: r** | **Approximate Mantel t-test** | **Bootstrap*** |
| --- | --- | --- | --- |
| **Only MPT**  **(119 characters, wing characters included)** | 0.63791 | 7.4916 | 3000, 0, 0 |
| **1st tree of 2 MPT**  **(106 characters, wing characters excluded)** | 0.53999 | 6.9837 | 3000, 0, 0 |
| **2nd tree of 2 MPT**  **(106 characters, wing characters excluded)** | 0.54107 | 6.8150 | 3000, 0, 0 |
