# Supplementary figures and images for: Evolutionary Constraints in Hind Wing Shape in Chinese Dung Beetles (Coleoptera: Scarabaeinae)
Source: PLoS One. 2011 Jun 27;6(6):e21600. doi: 10.1371/journal.pone.0021600 (PMC3124545; doi:10.1371/journal.pone.0021600)

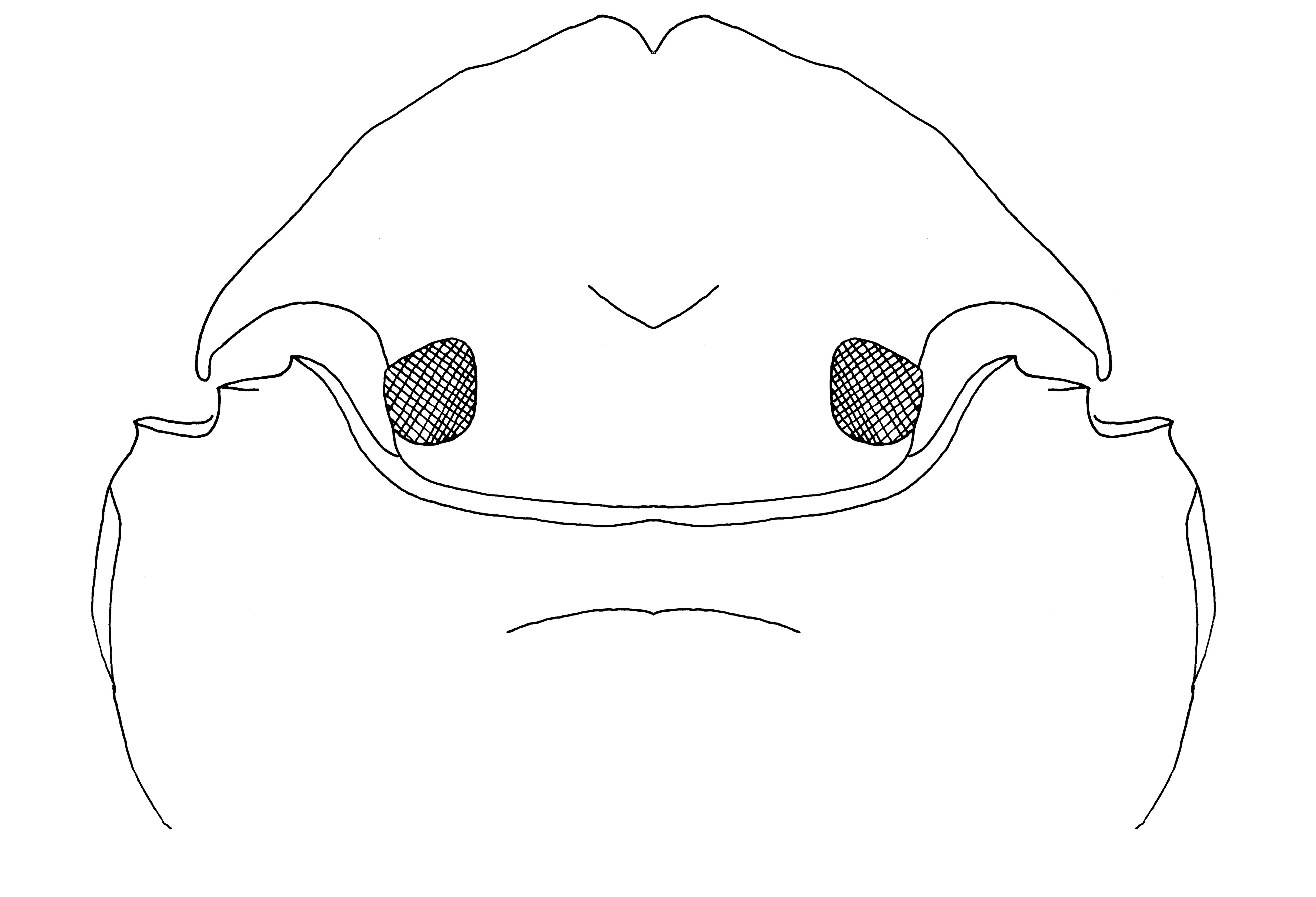

Supplement: Figure S1 — Head and pronotum ( Synapsis ). (TIF) [file pone.0021600.s006.tif]

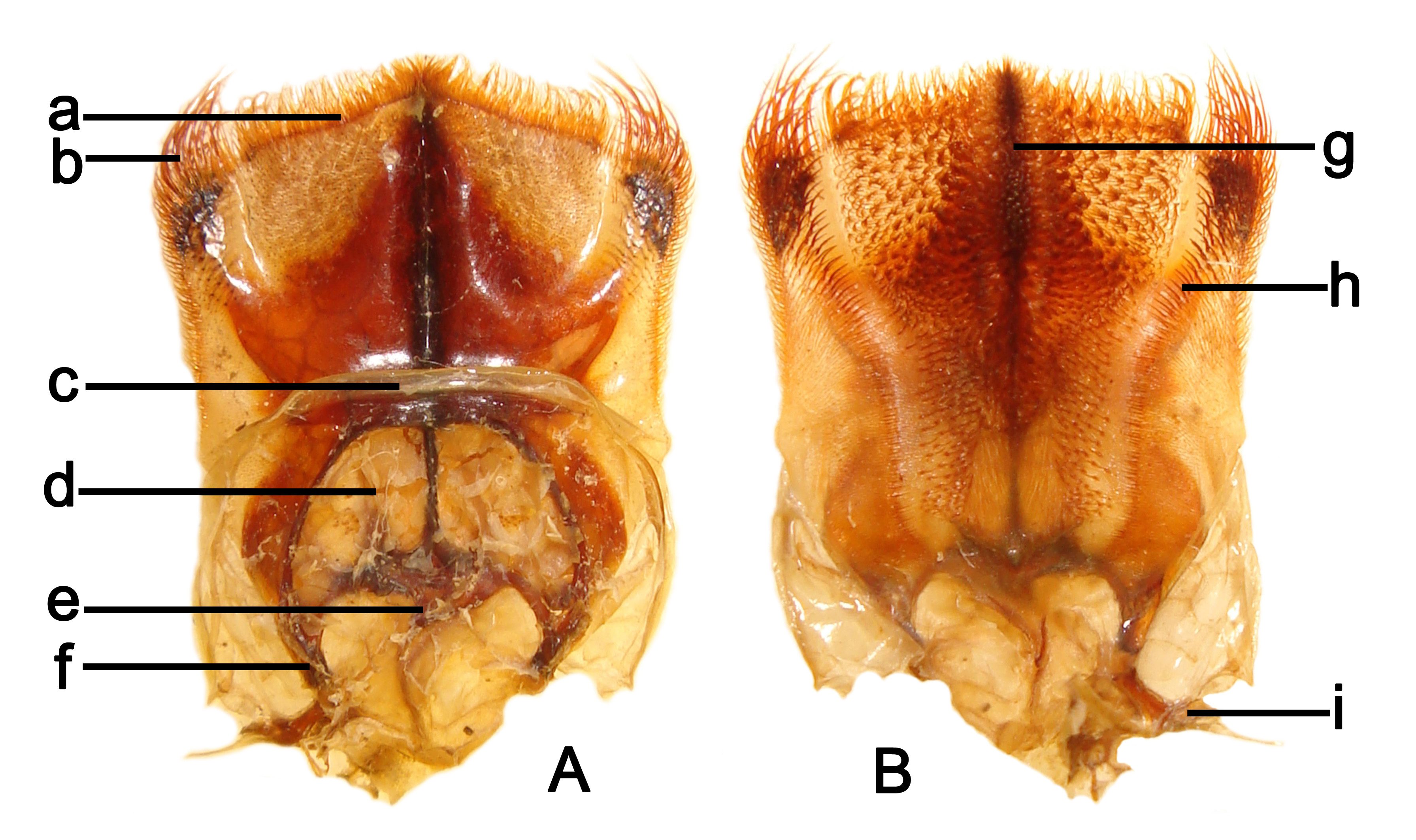

Supplement: Figure S2 — Epipharynx ( Heliocopris dominus Bates, 1868). (A) Dorsal view. (B) Ventral view. (a) Apical margin of distal epipharynx; (b) Fringe; (c) Clypeal-labral suture; (d) Cavity on dorsal side; (e) Median tormal process; (f) Lateral tormal process; (g) Anterior median ventral process; (h) Lateral combs; (i) Closed circles. (TIF) [file pone.0021600.s007.tif]

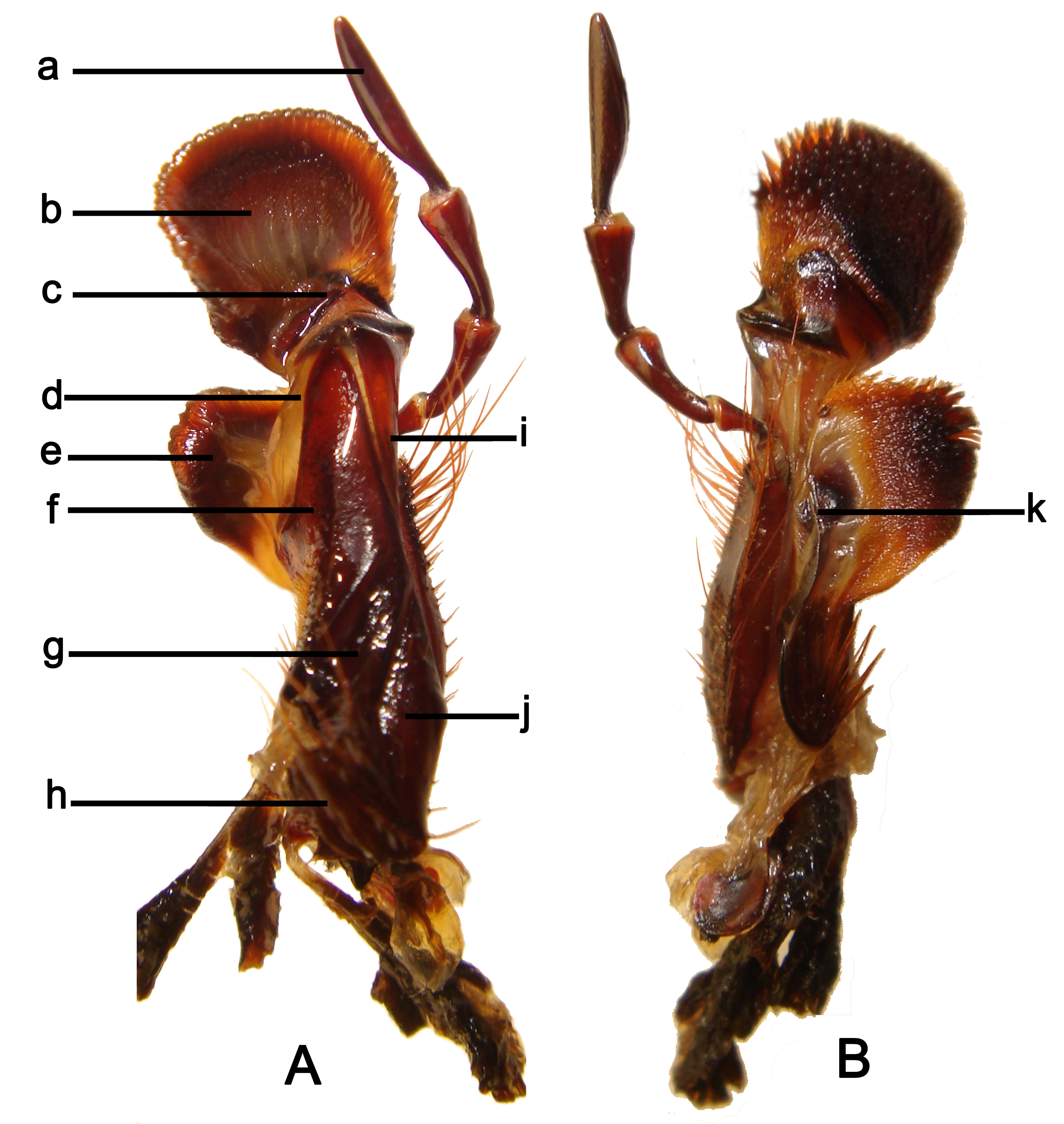

Supplement: Figure S3 — Maxilla ( Heliocopris dominus Bates, 1868). (A) Ventral view. (B) Dorsal view. (a) Maxillary palp; (b) Galea; (c) Sclerite of the galea; (d) Parastipes; (e) Lacinia; (f) Dististipes; (g) Basistipes; (h) Maxacoria; (i) Lateral sclerite; (j) Cardo; (k) Lacinial articulation sclerite. (TIF) [file pone.0021600.s008.tif]

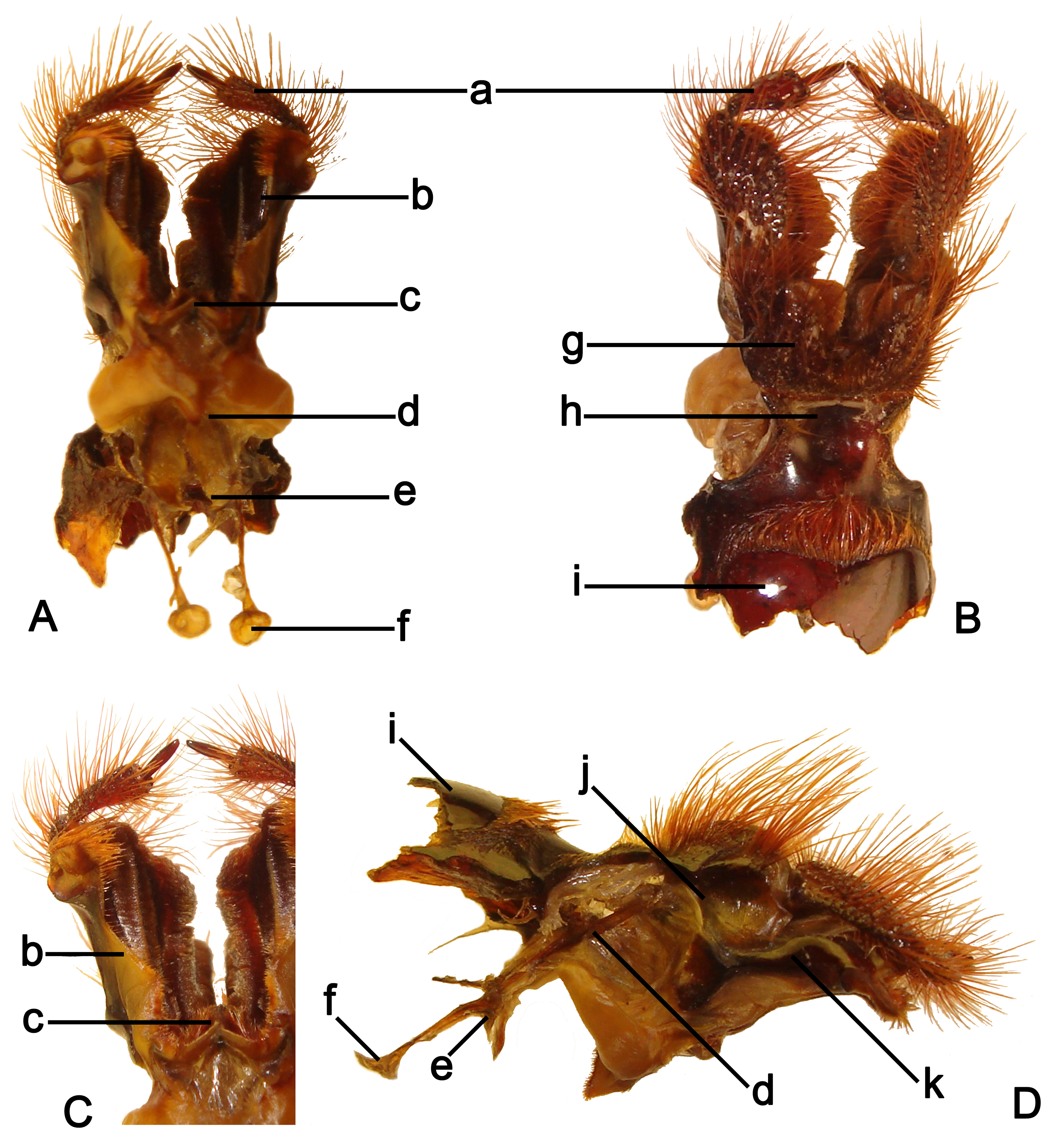

Supplement: Figure S4 — Labium ( Heliocopris dominus Bates, 1868). (A) Labial palpus. (B) Paraglossae. (C) Glossae. (D) Distal transverse bridge. (E) Proximal transverse bridge. (F) Apodemes. (G) Mentum. (H) Submentum. (I) Gula. (J) Palpomere strut. (K) Paraglossal strut. (TIF) [file pone.0021600.s009.tif]

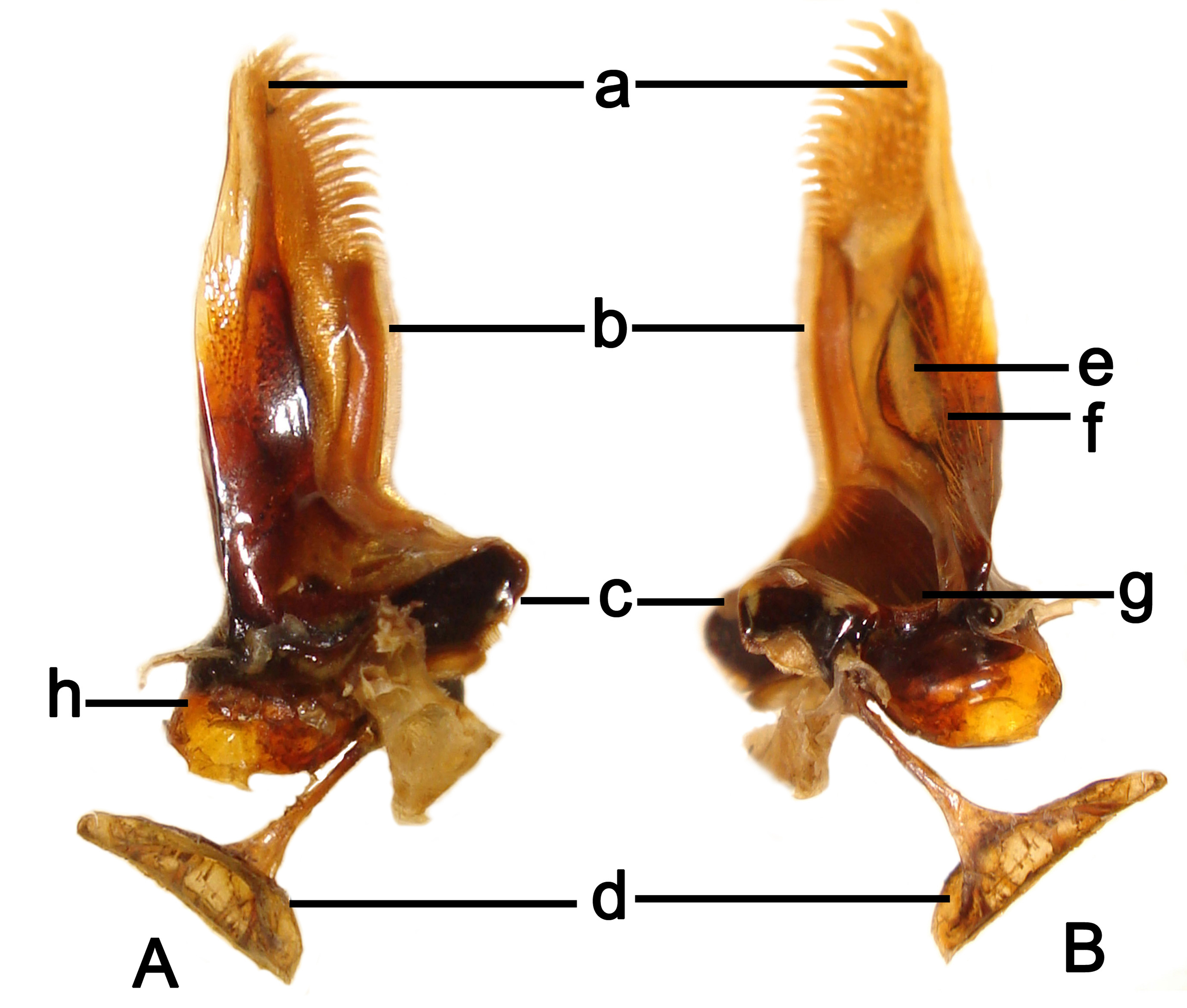

Supplement: Figure S5 — Mandibles ( Heliocopris dominus Bates, 1868). (A) Right mandible ventral view. (B) Right mandible dorsal view. (a) Incisor; (b) Prostheca; (c) Molar lobe; (d) Apodemes; (e) Incisor lobe; (f) Longitudinal carina; (g) Conjunctivus; (h) Receptacle. (TIF) [file pone.0021600.s010.tif]

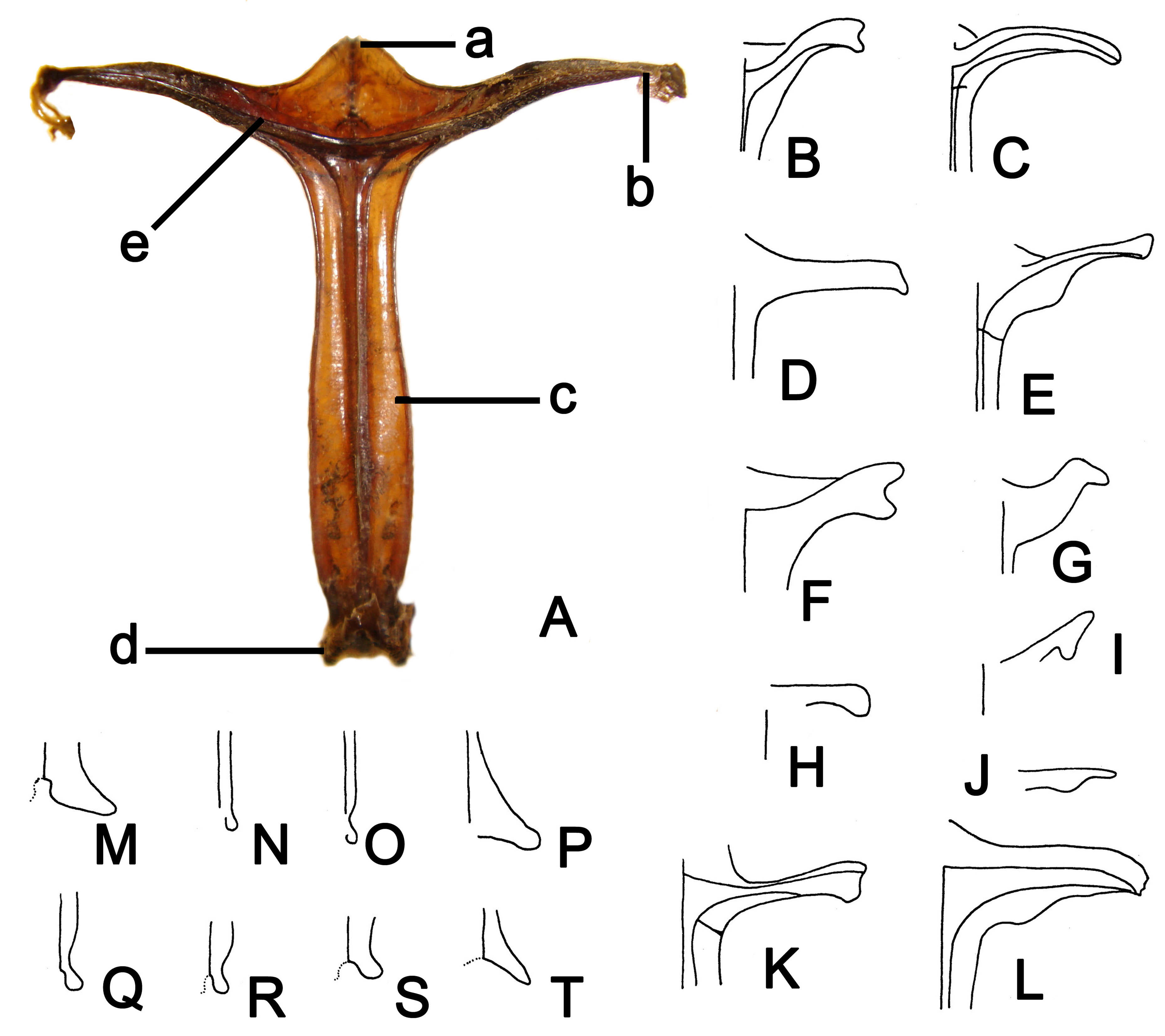

Supplement: Figure S6 — Metendosternite, dorsal view. (A) Heliocopris dominus Bates, 1868. (B) Eurysternus. (C) Dichotomius. (D) Glyphoderus. (E) Garreta. (F) Anachalcos. (G) Cyptochirus. (H) Garreta. (I) Tragiscus. (J) Canthon. (K) Copris. (L) Kheper. (M) Gymnopleurus. (N) Epirinus. (O) Sulcophanaeus. (P) Scaptocnemis. (Q) Glyphoderus. (R) Anachalcos. (S) Kheper. (T) Garreta. (B–T from Philips et al., 2004). (a) Frontal triangle; (b) Furcal arm; (c) Main body; (d) End of main body; (e) Lateral chitinous line in furcal arms. (TIF) [file pone.0021600.s011.tif]

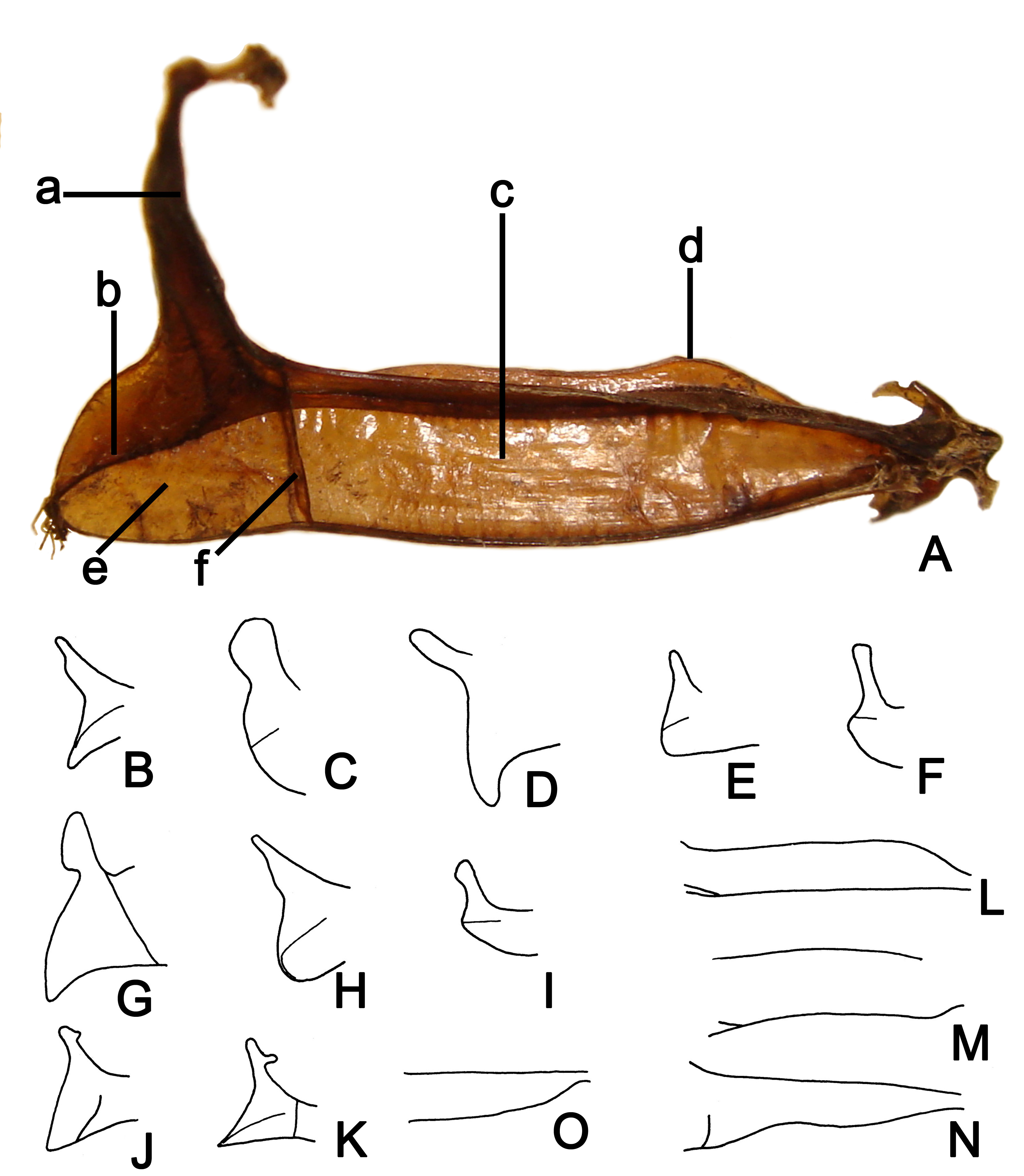

Supplement: Figure S7 — Metendosternite, lateral view. (A) Heliocopris dominus Bates, 1868. (B) Tragiscus. (C) Circellium. (D) Eurysternus. (E) Phanaeus. (F) Glyphoderus. (G) Kheper. (H) Anachalcos. (I) Anomiopsoides. (J) Garreta. (K) Canthon. (L) Anachalcos. (M) Eurysternus. (N) Onthophagus. (O) Garreta. (B–O from Philips et al., 2004). (a) Furcal arm; (b) Frontal midline; (c) Posterior attachment; (d) Midline; (d) Frontal triangle; (f) Lateral chitinous projection. (TIF) [file pone.0021600.s012.tif]

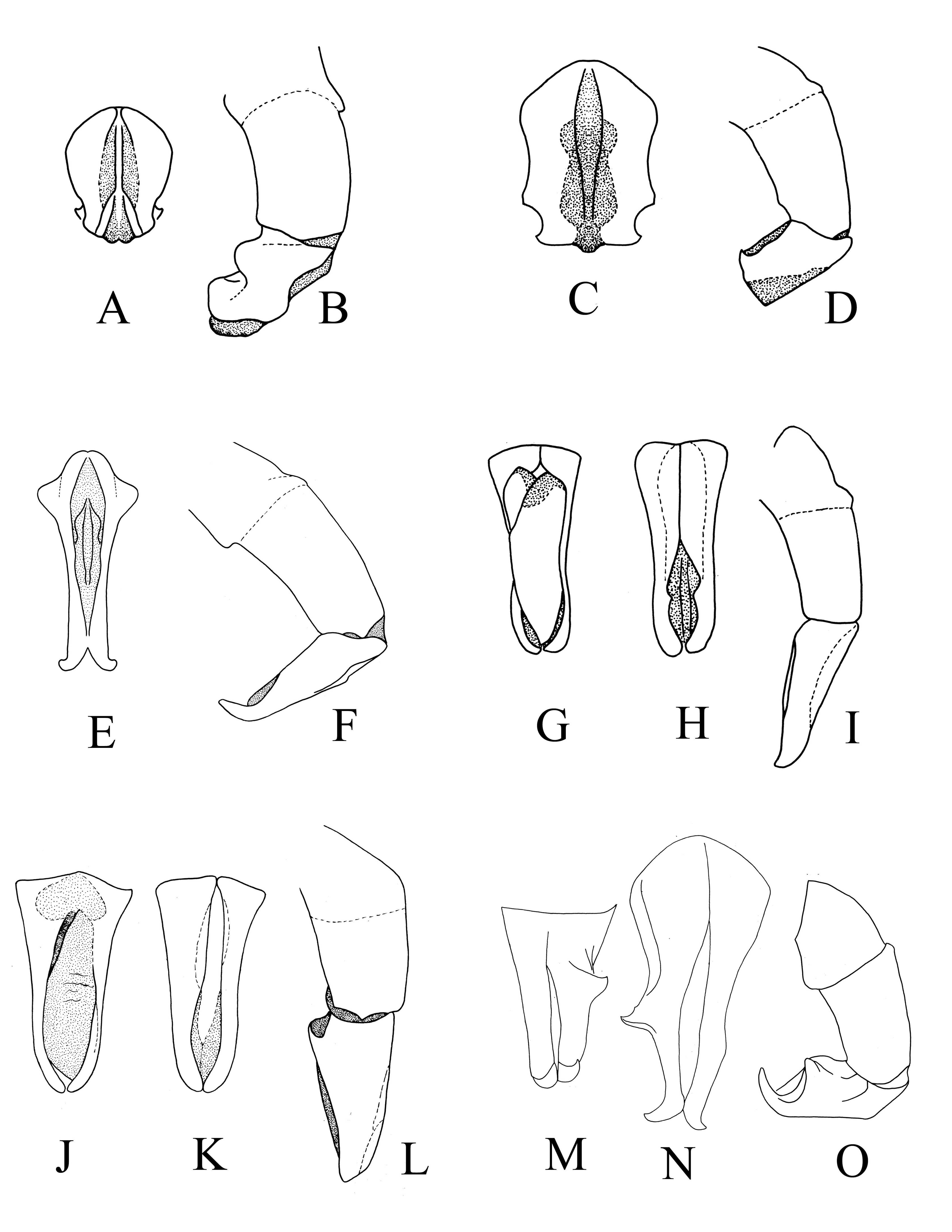

Supplement: Figure S8 — Aedeagus. (A–B) Liatongus bucerus. (C–D) Onthophagus (Macronthophagus) diabolicus. (E–F) Euonthophagus amyntas. (G–I) Copris szechouanicus. (J–L) Heliocopris bucephalus. (M–O) Scarabaeus babori. (Ventral view: G, J, M; Dorsal view: A, C, E, H, K, N; Lateral view: B, D, F, I, L, O.). (TIF) [file pone.0021600.s013.tif]

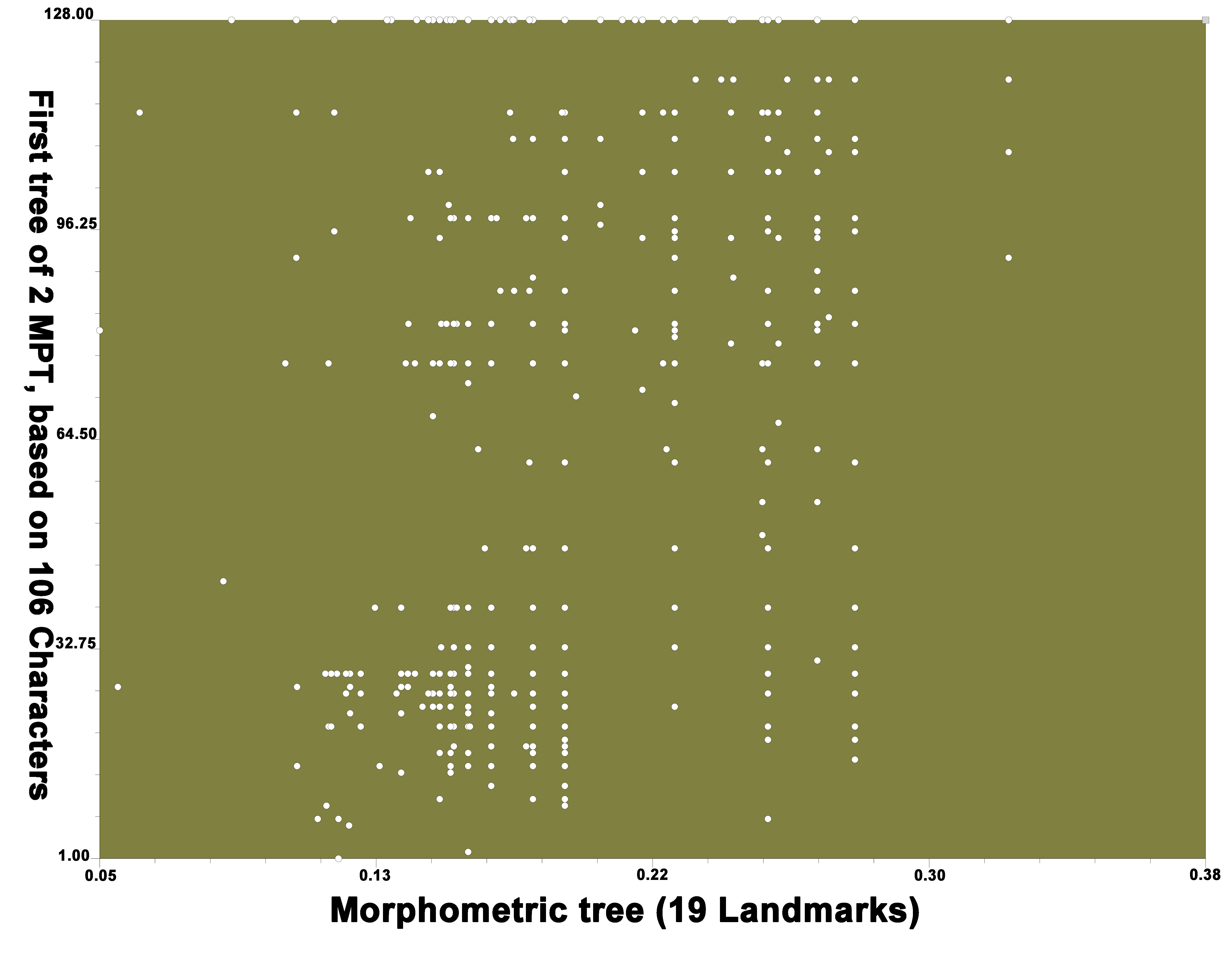

Supplement: Figure S11 — Correlation analysis of morphometric tree and first of the two most parsimonious phylogenetic trees. Matrix correlation: r = 0.53999, significantly correlated at the 1% level. (TIF) [file pone.0021600.s016.tif]
